# Incidence Trends of Colorectal Cancer by Social and Behavioral Factors—A Pooled Cohort Study

**DOI:** 10.1002/ijc.70656

**Published:** 2026-08-05

**Authors:** Emilia Muhonen, Karri Seppä, Heidi Ryynänen, Johan G. Eriksson, Tommi Härkänen, Pekka Jousilahti, Paul Knekt, Seppo Koskinen, Satu Männistö, Ossi Rahkonen, Maarit A. Laaksonen, Janne Pitkäniemi, Sanna Lappi‐Heikkinen, METCA Study Group

**Affiliations:** ^1^ Institute for Statistical and Epidemiological Cancer Research Finnish Cancer Registry Helsinki Finland; ^2^ Department of Public Health University of Helsinki Helsinki Finland; ^3^ Department of Public Health Finnish Institute for Health and Welfare (THL) Helsinki Finland; ^4^ Department of General Practice and Primary Health Care University of Helsinki and Helsinki University Hospital Helsinki Finland; ^5^ Folkhälsan Research Center Helsinki Finland; ^6^ School of Public Health, Faculty of Medicine University of Sydney Sydney Australia; ^7^ Faculty of Social Sciences, Health Sciences Tampere University Tampere Finland

**Keywords:** cohort study, colorectal cancer, incidence trend, risk factors

## Abstract

Colorectal cancer (CRC) represents more than 10% of the global cancer burden. Unlike in some other cancers, no single risk factor accounts for most CRC cases. Despite the well‐studied overall incidence trend of CRC, little information is available on risk factor‐specific incidence trends of CRC. This study aims to evaluate the rarely studied incidence trends of CRC in males and females with variable levels of exposure to specific risk factors, including education level, cigarette smoking, alcohol consumption, physical inactivity, and body mass index (BMI). We used Finnish pooled cohort data from seven cohorts linked to the population‐based Finnish Cancer Registry. The pooled data included 224,048 subjects, of whom 2006 were diagnosed with CRC. CRC incidence adjusted for known risk factors increased annually by 1.7% in males and 1.0% in females. In risk factor‐stratified analysis, the average annual percentage increase in CRC incidence was especially high in current male smokers. This study further strengthens the evidence that living with overweight or obesity, having a tall body height, or living with diabetes mellitus (DM) associates with an increased risk of CRC. Cigarette smoking was associated with an increased CRC risk in males, while heavy alcohol consumption was associated with an increased risk in females. In this study, education level or physical inactivity were not statistically significant risk factors for CRC. Our findings highlight the need for effective public health strategies, with special emphasis on preventing overweight, obesity, and smoking along with other modifiable lifestyle factors known to affect CRC risk.

## What's New?

Although colorectal cancer (CRC) incidence is well characterized overall, risk factor‐specific incidence trends remain poorly understood. Here, to examine CRC incidence by lifestyle and clinical risk factors, the authors analyzed data from nearly 225,000 participants in seven Finnish population‐based cohorts. CRC incidence increased annually in both sexes, with the most pronounced rise observed among current male smokers. Smoking, heavy alcohol consumption, overweight or obesity, tall stature, and diabetes were associated with increased CRC risk. No association was detected for education level and physical inactivity. The findings reinforce the importance of targeted public health strategies, particularly smoking prevention, to reduce CRC burden.

## Graphical abstract

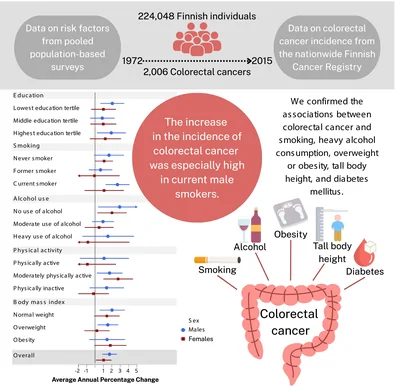

**Abbreviations**
AAPC: average annual percent change
ATH: Regional Health and Well‐being Study
BMI: body mass index
CI: confidence interval
CRC: colorectal cancer
DM: diabetes mellitus
FMCF: The Finnish Mobile Clinic Health Examination Follow‐up Study
H2000: Health 2000 Study
HBCS: Helsinki Birth Cohort Study
HHS: Helsinki Health Study
HR: hazard ratio
ICD‐10: International Classification of Diseases 10th revision
METCA: Prospective METa Cohort Study of Cancer Burden in Finland
MFH: Mini‐Finland Health Study
SES: socioeconomic status
THL: Finnish Institute for Health and Welfare
UI: uncertainty interval
WHO: World Health Organization

## 1 Introduction

Colorectal cancer (CRC) is the third most common cancer worldwide, representing around 10% of the global cancer burden in 2022 [1]. Globally, the age‐standardized incidence rate of CRC increased from 22.2 (95% uncertainty interval (UI): 21.3–23.0) per 100,000 persons to 26.7 (95% UI: 24.6–28.9) per 100,000 persons between 1990 and 2019 [2]. More than 1.9 million new cases of CRC, including anal cancer, emerged in 2022, and the incidence was higher in males than in females. The incidence rates of CRC are three to four times higher in economically transitioned countries compared with transitioning countries [1]. Notably, stabilizing or decreasing trends of CRC are seen in highly developed countries, while an increasing cancer burden is observed in countries in transition [3]. A systematic review and meta‐analysis conducted in 2024 found that CRC incidence was 11% higher and CRC‐caused mortality was 21% higher in subjects who had lower neighborhood socioeconomic status (SES) compared with those who had a higher neighborhood SES [4]. In Finland, the incidence rate of CRC was higher in individuals with higher education levels and higher SES compared with those with lower education levels and lower SES [5].

Unlike in some cancers, like lung cancer and skin melanoma, no single dominant risk factor accounts for most CRC cases [6]. Risk factors of CRC include male sex [6, 7], inflammatory bowel disease [7], diabetes mellitus (DM) [8, 9], and family history of CRC [7, 10]. Genetic, environmental, hormonal, and nutritional factors that lead to greater adult height are also considered to increase the CRC risk [6, 11, 12]. Lifestyle factors that increase the CRC risk include a high body mass index (BMI) [7, 10, 13, 14, 15] and a large waist circumference [15]. Sedentary behaviors associate with an increased risk of CRC [7], while physical activity may decrease the risk [10, 11, 16]. Other lifestyle factors that increase the CRC risk include tobacco smoking [7, 10, 13, 17, 18], alcohol consumption [7, 10, 11, 13, 17, 19], particularly alcohol intake above 30 g per day [11], and the consumption of processed and red meat [7, 11, 13, 19, 20, 21]. Lifestyle factors associated with a decreased risk include consumption of dietary fibers, especially from wholegrains [11, 19, 22], dietary and supplementary calcium [11, 22, 23], and dairy products [11, 19, 22].

Healthy food habits and diets following nutritional recommendations have become more common since 1979 in all regions of Finland. Concurrently, obesity has become more frequent in the population [24]. Meanwhile, the consumption of fruits and vegetables has increased in both males and females, while the consumption of red and processed meat has decreased in males [25]. Still, according to a 2018 report, only 14% of Finnish males and 22% of Finnish females met the recommendations for fruit and vegetable intake, whereas 21% of Finnish males and 74% of Finnish females met those for red and processed meat intake [26]. In 2017, 67% of Finnish males and 76% of Finnish females consumed less dietary fiber than recommended [26]. The underlying causes of obesity have changed due to increased consumption of ultra‐processed foods [27, 28] and increased sedentary behavior [29]. Further, patterns of smoking and alcohol consumption with respect to cigarette contents [30] and types of alcoholic drinks [31] have changed.

Despite the well‐studied overall incidence trend of CRC, little information is available on risk factor‐specific incidence trends of CRC. Our study aims to evaluate the rarely studied incidence trends of CRC in males and females with variable exposure levels to specific risk factors, including education level, cigarette smoking, alcohol consumption, physical inactivity, and BMI.

## 2 Materials and Methods

We used the METCA (Prospective METa Cohort Study of Cancer Burden in Finland) project, which pools data from seven health studies in Finland between 1972 and 2015 [32]. It included one cohort from the University of Helsinki: Helsinki Health Study (HHS, 2000–2002, end of follow‐up in 2015) [33], and six cohorts from the Finnish Institute for Health and Welfare (THL): The Finnish Mobile Clinic Health Examination Follow‐up Study (FMCF, 1973–1976, end of follow‐up in 2013) [34], National FINRISK Study (comprising nine studies conducted every 5 years from 1972 to 2012, end of follow‐up in 2013) [35], Mini‐Finland Health Survey (MFH, 1978–1980, end of follow‐up in 2013) [34], Health 2000 Study (H2000, 2000–2001, end of follow‐up in 2015) [36], Helsinki Birth Cohort Study (HBCS, 2000, end of follow‐up in 2013) [37], and Regional Health and Well‐being Study (ATH) [38]. The ATH study consisted of two datasets: ATH1 and ATH2. ATH1 was collected in 2010–2011 (end of follow‐up in 2015) and ATH2 in 2012–2015 (end of follow‐up in 2015) [32, 38]. Data requirements for the health studies included in the METCA project were: having information on the known or suspected cancer risk factors and having a national personal identity code available to link both cancer and vital status information individually [32]. Study subject inclusion was based on some variant of random selection from the Finnish population in all cohorts except for HBCS, which is a birth cohort [32]. After the exclusion of subjects diagnosed with CRC before the beginning of the follow‐up, the pooled data included 224,048 subjects, of whom 2006 were diagnosed with CRC. About 45% (*N* = 100,522) of the subjects were males and 55% (*N* = 123,526) were females. Most of the studies had been validated earlier, with validation results presented in the cohort profiles of each study [34, 35, 36]. Heterogeneity in cancer associations between the studies has been assessed by Pitkäniemi et al. for colorectal and lung cancer [32].

We extracted and harmonized information on education levels, cigarette smoking, alcohol consumption, physical activity, BMI, height, and DM. Information on education levels, cigarette smoking, alcohol consumption, physical activity, and DM had been collected using self‐reported questionnaires in all cohorts from THL. Body weight and height had been measured clinically in all other cohorts from THL (FMCF, FINRISK, MFH, H2000, and HBCS) except for ATH, which collected information on these exposures using questionnaires alone. Information on sex, birth year, and age at baseline was registry‐based information in all cohorts from THL [32]. In HHS, information on exposures had been collected using self‐reported questionnaires. Additionally, in HHS, information on exposures had been collected during health examinations for those subjects who agreed to participate in these examinations [33]. Sufficiency and consistency over various cohorts were requirements for cohort inclusion, and as close a match as possible was sought in terms of the survey questions for each considered variable in each cohort [32]. We handled missing covariates as a separate category in all analyses.

We classified education levels as low (reference group), middle, or high. Classification was made according to tertiles of the number of years of schooling by sex and birth cohort (eight cohorts by the year of birth: < 1920, 10‐year periods from 1920 to 1979, and 1980 or later) to overcome changing secular trends [32]. Subjects who had never smoked cigarettes (HBCS) or never smoked cigarettes regularly (FMCF, FINRISK, MFH, H2000, ATH) were classified as “never smokers,” subjects who reported having smoked cigarettes regularly but not at the time of the health survey were classified as “former smokers,” and subjects who reported smoking cigarettes regularly were classified as “smokers” [32, 39]. Subjects who reported using 0 g of alcohol per week (MFH) or per month (FMCF), never using alcohol (FINRISK, H2000, HBCS), or not using alcohol within the past year (ATH) were classified as “non‐users” (reference group) [32]. Alcohol use of less than 7 (females) or 14 (males) units per week was classified as “moderate use,” and alcohol use of 7 (females) or 14 (males) units or more per week as “heavy use.” Subjects who reported exercises maintaining physical fitness (e.g., jogging, swimming, running, ball games, heavy gardening, competitive sports) were classified as “active” (reference group), subjects who reported light physical activities (e.g., walking, cycling, light housework) during leisure time were classified as “moderately active,” and subjects who reported 0 times of moderate physical activity per week (HBCS) or not engaging in physically burdening activities or hobbies during leisure time (FMCF, FINRISK, MFH, H2000, ATH) were classified as “inactive” [32, 40].

BMI was calculated with the formula weight (kilograms)/height (meters) squared (kg/m^2^) from clinically measured or self‐reported weight and height [32]. Subjects with a BMI of less than 25 kg/m^2^ were considered the reference group. Subjects with a BMI of 25 kg/m^2^ or over but less than 30 kg/m^2^ were considered as living with overweight, and subjects with a BMI of 30 kg/m^2^ or over were considered as living with obesity [41]. Body height (measured or self‐reported) was classified in tertiles by sex and birth cohort (eight cohorts by the year of birth: before 1920, periods of 10 years from 1920 to 1979, and 1980 or later), with increasing cut‐off points for tertiles toward the later birth cohorts [32]. The exact categorization is given in Table S1. For males born before 1920, those 168 cm or shorter were classified into the shortest tertile (reference group) and those taller than 173 cm into the tallest tertile. For males born in or after 1980, the respective cut‐off points were 176 cm (reference group) and 182 cm. For females born before 1920, those 154 cm or shorter were classified into the shortest tertile (reference group) and those taller than 159 cm into the tallest tertile. For females born in or after 1980, the respective cut‐off points were 163 cm (reference group) and 168 cm.

We used the personal identity code when linking the survey data to the population‐based Finnish Cancer Registry at the individual level. Linking to the registry data was performed by owners of the study survey data, and the analyses of the pooled data were conducted using pseudonymized data. The Finnish Cancer Registry holds national cancer data of high coverage and accuracy since 1953 [42]. We retrieved incident CRCs for the International Classification of Diseases 10th revision (ICD‐10) codes C18.0–C20.99 from the cancer registry. The follow‐up started at the time of the individual health survey and ended at the date of CRC diagnosis, death, or the end of the follow‐up (in 2013–2015, depending on the study).

## 3 Statistics

Age‐standardized incidence rates of CRC were calculated by risk factors over the entire period 1972–2015 and separately for five calendar periods (decades from 1972–1981 to 2002–2011 and a 4‐year period of 2012–2015). We used the World Health Organization's (WHO) World Standard Population for 2000–2025 in 14 age groups (18–24 years, 5‐year groups from 25–29 to 80–84 and ≥ 85 years) as weights in the standardization. We modeled both the effects of risk factors and the effects of calendar periods on cancer incidence using Poisson regression with log person‐years as the offset, where the cause‐specific hazard of CRC is the product of the baseline hazard and the corresponding hazard ratios (HRs). The HRs were based on Model 1, which included the effects of age and calendar period (the same categorization as above) and their interactions, and the main effect of time since survey (< 10 and ≥ 10 years). In addition, we estimated the average annual percent change (AAPC) based on Model 2, which included the main effects of age, calendar year, and time since survey. The effects of risk factors and the AAPC were estimated both by adjusting and without adjusting for the effects of other risk factors (education level, cigarette smoking, alcohol consumption, physical activity, BMI, height, and DM). We used the likelihood ratio test to assess the statistical significance of AAPC and heterogeneity in the AAPCs between risk factor groups. The heterogeneity tests were based on models that also included the interaction between age and risk factor groups. Information on alcohol consumption was not available from the FINRISK cohorts in 1972, 1977, and 1982, or from the HBCS cohort, and physical activity or height measurements were not available from the FMCF and MFH cohorts (“missing by protocol”). The corresponding covariates from these cohorts were treated as a separate missing category in the risk factor analysis. To assess the effect of potential exposure misclassification in longer follow‐ups, we performed a sensitivity analysis restricting the follow‐up to the first 10 and 15 years after the survey. Heterogeneity of the studies was partially accounted for in the analyses by allowing the baseline hazard of cancer to vary by calendar period and follow‐up time.

## 4 Results

CRC occurred in 1020 (1.0%) males and in 986 (0.8%) females (Table 1). Around one in three (32.3%) males reported current smoking, while 18.3% of females reported current smoking. Fewer males (12.5%) reported alcohol abstinence compared with females (23.4%). It should, however, be noted that information on alcohol use was missing by protocol for 16.5% of males and 14.1% of females. A larger proportion of males (18.0%) reported themselves as physically active compared with females (14.0%), while more females (47.4%) reported being moderately active compared with males (44.0%). Information on physical activity was missing by protocol for 13.1% of males and 10.9% of females. Nearly half (43.7%) of the males and one in three (31.9%) females were living with overweight, while the proportions of males and females living with obesity were 15.8% and 18.3%, respectively.

**TABLE 1:** Number and proportion of subjects by age, periods, and risk factors stratified by sex.

Males						
	1972–1981 (*N* = 24,568)	1982–1991 (*N* = 7150)	1992–2001 (*N* = 12,392)	2002–2011 (*N* = 14,013)	2012–2015 (*N* = 42,399)	Total (*N* = 100,522)
Age group						
18–24	1291 (5.3%)	114 (1.6%)	540 (4.4%)	373 (2.7%)	1625 (3.8%)	3943 (3.9%)
25–29	2801 (11.4%)	879 (12.3%)	950 (7.7%)	899 (6.4%)	2112 (5.0%)	7641 (7.6%)
30–34	3141 (12.8%)	1042 (14.6%)	1034 (8.3%)	954 (6.8%)	2406 (5.7%)	8577 (8.5%)
35–39	3036 (12.4%)	942 (13.2%)	1208 (9.7%)	1062 (7.6%)	2422 (5.7%)	8670 (8.6%)
40–44	3218 (13.1%)	848 (11.9%)	1389 (11.2%)	1230 (8.8%)	2406 (5.7%)	9091 (9.0%)
45–49	3151 (12.8%)	881 (12.3%)	1401 (11.3%)	1283 (9.2%)	2849 (6.7%)	9565 (9.5%)
50–54	2694 (11.0%)	913 (12.8%)	1340 (10.8%)	1573 (11.2%)	3375 (8.0%)	9895 (9.8%)
55–59	2205 (9.0%)	946 (13.2%)	1900 (15.3%)	1609 (11.5%)	4003 (9.4%)	10,663 (10.6%)
60–64	1403 (5.7%)	585 (8.2%)	1227 (9.9%)	1609 (11.5%)	4709 (11.1%)	9533 (9.5%)
65–69	797 (3.2%)	0 (0.0%)	681 (5.5%)	1160 (8.3%)	4951 (11.7%)	7589 (7.5%)
70–74	483 (2.0%)	0 (0.0%)	436 (3.5%)	802 (5.7%)	3094 (7.3%)	4815 (4.8%)
75–79	232 (0.9%)	0 (0.0%)	107 (0.9%)	772 (5.5%)	4368 (10.3%)	5479 (5.5%)
80–84	89 (0.4%)	0 (0.0%)	106 (0.9%)	485 (3.5%)	2623 (6.2%)	3303 (3.3%)
85+	27 (0.1%)	0 (0.0%)	73 (0.6%)	202 (1.4%)	1456 (3.4%)	1758 (1.7%)
Education						
Low	10,529 (42.9%)	3712 (51.9%)	5108 (41.2%)	5640 (40.2%)	15,408 (36.3%)	40,397 (40.2%)
Middle	8997 (36.6%)	1859 (26.0%)	3925 (31.7%)	4121 (29.4%)	12,254 (28.9%)	31,156 (31.0%)
High	4769 (19.4%)	1348 (18.9%)	3207 (25.9%)	3833 (27.4%)	12,618 (29.8%)	25,775 (25.6%)
Missing	273 (1.1%)	231 (3.2%)	152 (1.2%)	419 (3.0%)	2119 (5.0%)	3194 (3.2%)
Missing by protocol	0 (0.0%)	0 (0.0%)	0 (0.0%)	0 (0.0%)	0 (0.0%)	0 (0.0%)
Smoking						
Never smoker	6008 (24.5%)	1660 (23.2%)	3882 (31.3%)	4513 (32.2%)	15,680 (37.0%)	31,743 (31.6%)
Former smoker	6565 (26.7%)	2495 (34.9%)	3950 (31.9%)	4976 (35.5%)	16,245 (38.3%)	34,231 (34.1%)
Current smoker	11,875 (48.3%)	2980 (41.7%)	4446 (35.9%)	4195 (29.9%)	9008 (21.2%)	32,504 (32.3%)
Missing	120 (0.5%)	15 (0.2%)	114 (0.9%)	329 (2.3%)	1466 (3.5%)	2044 (2.0%)
Missing by protocol	0 (0.0%)	0 (0.0%)	0 (0.0%)	0 (0.0%)	0 (0.0%)	0 (0.0%)
Alcohol						
No use	3367 (13.7%)	161 (2.3%)	624 (5.0%)	1501 (10.7%)	6862 (16.2%)	12,515 (12.5%)
Moderate use	7939 (32.3%)	2308 (32.3%)	7755 (62.6%)	9811 (70.0%)	28,014 (66.1%)	55,827 (55.5%)
Heavy use	1834 (7.5%)	299 (4.2%)	1912 (15.4%)	2266 (16.2%)	5439 (12.8%)	11,750 (11.7%)
Missing	58 (0.2%)	65 (0.9%)	1175 (9.5%)	435 (3.1%)	2084 (4.9%)	3817 (3.8%)
Missing by protocol	11,370 (46.3%)	4317 (60.4%)	926 (7.5%)	0 (0.0%)	0 (0.0%)	16,613 (16.5%)
Physical activity						
Active	1780 (7.2%)	1394 (19.5%)	2836 (22.9%)	3028 (21.6%)	9086 (21.4%)	18,124 (18.0%)
Moderately active	5830 (23.7%)	3447 (48.2%)	6447 (52.0%)	7220 (51.5%)	21,290 (50.2%)	44,234 (44.0%)
Inactive	3587 (14.6%)	2127 (29.7%)	2523 (20.4%)	3349 (23.9%)	10,658 (25.1%)	22,244 (22.1%)
Missing	173 (0.7%)	182 (2.5%)	586 (4.7%)	416 (3.0%)	1365 (3.2%)	2722 (2.7%)
Missing by protocol	13,198 (53.7%)	0 (0.0%)	0 (0.0%)	0 (0.0%)	0 (0.0%)	13,198 (13.1%)
BMI						
Normal weight	11,464 (46.7%)	2639 (36.9%)	3690 (29.8%)	4634 (33.1%)	14,851 (35.0%)	37,278 (37.1%)
Overweight	10,226 (41.6%)	3262 (45.6%)	5235 (42.2%)	6482 (46.3%)	18,726 (44.2%)	43,931 (43.7%)
Obesity	2467 (10.0%)	1118 (15.6%)	2100 (16.9%)	2685 (19.2%)	7505 (17.7%)	15,875 (15.8%)
Missing	411 (1.7%)	131 (1.8%)	1367 (11.0%)	212 (1.5%)	1317 (3.1%)	3438 (3.4%)
Missing by protocol	0 (0.0%)	0 (0.0%)	0 (0.0%)	0 (0.0%)	0 (0.0%)	0 (0.0%)
Height (cm)						
Mean (SD)	172 (6.7)	174 (6.8)	176 (6.9)	176 (7.0)	177 (6.9)	176 (7.1)
Range	123–204	135–198	134–204	122–218	100–210	100–218
Missing	13,600	129	1359	174	1147	16,409
Height (tertiles)						
Shortest tertile	5096 (20.7%)	2935 (41.0%)	3780 (30.5%)	5354 (38.2%)	14,189 (33.5%)	31,354 (31.2%)
Middle tertile	3433 (14.0%)	2171 (30.4%)	3608 (29.1%)	4593 (32.8%)	13,715 (32.3%)	27,520 (27.4%)
Tallest tertile	2439 (9.9%)	1915 (26.8%)	3645 (29.4%)	3892 (27.8%)	13,348 (31.5%)	25,239 (25.1%)
Missing	402 (1.6%)	129 (1.8%)	1359 (11.0%)	174 (1.2%)	1147 (2.7%)	3211 (3.2%)
Missing by protocol	13,198 (53.7%)	0 (0.0%)	0 (0.0%)	0 (0.0%)	0 (0.0%)	13,198 (13.1%)
Diabetes						
No	23,942 (97.5%)	6814 (95.3%)	11,626 (93.8%)	11,522 (82.2%)	33,718 (79.5%)	87,622 (87.2%)
Yes	545 (2.2%)	208 (2.9%)	526 (4.2%)	1073 (7.7%)	4999 (11.8%)	7351 (7.3%)
Missing	81 (0.3%)	128 (1.8%)	240 (1.9%)	1418 (10.1%)	3682 (8.7%)	5549 (5.5%)
Missing by protocol	0 (0.0%)	0 (0.0%)	0 (0.0%)	0 (0.0%)	0 (0.0%)	0 (0.0%)
Colorectal cancer						
Colorectal cancer	542 (2.2%)	124 (1.7%)	167 (1.3%)	94 (0.7%)	93 (0.2%)	1020 (1.0%)
No colorectal cancer	24,026 (97.8%)	7026 (98.3%)	12,225 (98.7%)	13,919 (99.3%)	42,306 (99.8%)	99,502 (99.0%)
Cohort						
ATH1	0 (0.0%)	0 (0.0%)	0 (0.0%)	6970 (49.7%)	0 (0.0%)	6970 (6.9%)
ATH2	0 (0.0%)	0 (0.0%)	0 (0.0%)	0 (0.0%)	39,757 (93.8%)	39,757 (39.6%)
FMCF	9883 (40.2%)	0 (0.0%)	0 (0.0%)	0 (0.0%)	0 (0.0%)	9883 (9.8%)
FINRISK	11,370 (46.3%)	7150 (100.0%)	6580 (53.1%)	6589 (47.0%)	2642 (6.2%)	34,331 (34.2%)
H2000	0 (0.0%)	0 (0.0%)	3924 (31.7%)	0 (0.0%)	0 (0.0%)	3924 (3.9%)
HBCS	0 (0.0%)	0 (0.0%)	926 (7.5%)	0 (0.0%)	0 (0.0%)	926 (0.9%)
HHS	0 (0.0%)	0 (0.0%)	962 (7.8%)	454 (3.2%)	0 (0.0%)	1416 (1.4%)
MFH	3315 (13.5%)	0 (0.0%)	0 (0.0%)	0 (0.0%)	0 (0.0%)	3315 (3.3%)

Females						
	1972–1981 (*N* = 25,430)	1982–1991 (*N* = 7482)	1992–2001 (*N* = 16,059)	2002–2011 (*N* = 18,484)	2012–2015 (*N* = 56,071)	Total (*N* = 123,526)
Age group						
18–24	1201 (4.7%)	151 (2.0%)	548 (3.4%)	614 (3.3%)	2542 (4.5%)	5056 (4.1%)
25–29	2526 (9.9%)	933 (12.5%)	1074 (6.7%)	1309 (7.1%)	3077 (5.5%)	8919 (7.2%)
30–34	3095 (12.2%)	991 (13.2%)	1212 (7.5%)	1230 (6.7%)	3195 (5.7%)	9723 (7.9%)
35–39	2939 (11.6%)	952 (12.7%)	1711 (10.7%)	1483 (8.0%)	3197 (5.7%)	10,282 (8.3%)
40–44	2973 (11.7%)	839 (11.2%)	2074 (12.9%)	1767 (9.6%)	3106 (5.5%)	10,759 (8.7%)
45–49	3099 (12.2%)	938 (12.5%)	2002 (12.5%)	1861 (10.1%)	3760 (6.7%)	11,660 (9.4%)
50–54	3054 (12.0%)	933 (12.5%)	1958 (12.2%)	2019 (10.9%)	4442 (7.9%)	12,406 (10.0%)
55–59	2587 (10.2%)	966 (12.9%)	2415 (15.0%)	1945 (10.5%)	4927 (8.8%)	12,840 (10.4%)
60–64	1613 (6.3%)	779 (10.4%)	1417 (8.8%)	1708 (9.2%)	5570 (9.9%)	11,087 (9.0%)
65–69	1031 (4.1%)	0 (0.0%)	505 (3.1%)	1249 (6.8%)	5599 (10.0%)	8384 (6.8%)
70–74	723 (2.8%)	0 (0.0%)	412 (2.6%)	935 (5.1%)	3800 (6.8%)	5870 (4.8%)
75–79	405 (1.6%)	0 (0.0%)	200 (1.2%)	1069 (5.8%)	5781 (10.3%)	7455 (6.0%)
80–84	146 (0.6%)	0 (0.0%)	280 (1.7%)	771 (4.2%)	4073 (7.3%)	5270 (4.3%)
85+	38 (0.1%)	0 (0.0%)	251 (1.6%)	524 (2.8%)	3002 (5.4%)	3815 (3.1%)
Education						
Low	11,206 (44.1%)	3517 (47.0%)	6148 (38.3%)	6907 (37.4%)	19,676 (35.1%)	47,454 (38.4%)
Middle	8706 (34.2%)	2088 (27.9%)	5850 (36.4%)	6061 (32.8%)	16,932 (30.2%)	39,637 (32.1%)
High	5244 (20.6%)	1569 (21.0%)	3826 (23.8%)	4848 (26.2%)	16,449 (29.3%)	31,936 (25.9%)
Missing	274 (1.1%)	308 (4.1%)	235 (1.5%)	668 (3.6%)	3014 (5.4%)	4499 (3.6%)
Missing by protocol	0 (0.0%)	0 (0.0%)	0 (0.0%)	0 (0.0%)	0 (0.0%)	0 (0.0%)
Smoking						
Never smoker	18,973 (74.6%)	4519 (60.4%)	8864 (55.2%)	9772 (52.9%)	31,932 (56.9%)	74,060 (60.0%)
Former smoker	1925 (7.6%)	1321 (17.7%)	3010 (18.7%)	4121 (22.3%)	13,676 (24.4%)	24,053 (19.5%)
Current smoker	4408 (17.3%)	1596 (21.3%)	3917 (24.4%)	4060 (22.0%)	8589 (15.3%)	22,570 (18.3%)
Missing	124 (0.5%)	46 (0.6%)	268 (1.7%)	531 (2.9%)	1874 (3.3%)	2843 (2.3%)
Missing by protocol	0 (0.0%)	0 (0.0%)	0 (0.0%)	0 (0.0%)	0 (0.0%)	0 (0.0%)
Alcohol						
No use	8196 (32.2%)	622 (8.3%)	2095 (13.0%)	3397 (18.4%)	14,606 (26.0%)	28,916 (23.4%)
Moderate use	4875 (19.2%)	2222 (29.7%)	10,254 (63.9%)	11,991 (64.9%)	32,651 (58.2%)	61,993 (50.2%)
Heavy use	417 (1.6%)	164 (2.2%)	1650 (10.3%)	2394 (13.0%)	5899 (10.5%)	10,524 (8.5%)
Missing	30 (0.1%)	71 (0.9%)	989 (6.2%)	702 (3.8%)	2915 (5.2%)	4707 (3.8%)
Missing by protocol	11,912 (46.8%)	4403 (58.8%)	1071 (6.7%)	0 (0.0%)	0 (0.0%)	17,386 (14.1%)
Physical activity						
Active	1181 (4.6%)	897 (12.0%)	2477 (15.4%)	3049 (16.5%)	9696 (17.3%)	17,300 (14.0%)
Moderately active	4993 (19.6%)	3673 (49.1%)	9926 (61.8%)	10,506 (56.8%)	29,505 (52.6%)	58,603 (47.4%)
Inactive	5549 (21.8%)	2666 (35.6%)	3124 (19.5%)	4329 (23.4%)	15,081 (26.9%)	30,749 (24.9%)
Missing	189 (0.7%)	246 (3.3%)	532 (3.3%)	600 (3.2%)	1789 (3.2%)	3356 (2.7%)
Missing by protocol	13,518 (53.2%)	0 (0.0%)	0 (0.0%)	0 (0.0%)	0 (0.0%)	13,518 (10.9%)
BMI						
Normal weight	12,290 (48.3%)	3643 (48.7%)	6871 (42.8%)	8464 (45.8%)	25,439 (45.4%)	56,707 (45.9%)
Overweight	8328 (32.7%)	2403 (32.1%)	4757 (29.6%)	6076 (32.9%)	17,874 (31.9%)	39,438 (31.9%)
Obesity	4423 (17.4%)	1333 (17.8%)	2824 (17.6%)	3599 (19.5%)	10,487 (18.7%)	22,666 (18.3%)
Missing	389 (1.5%)	103 (1.4%)	1607 (10.0%)	345 (1.9%)	2271 (4.1%)	4715 (3.8%)
Missing by protocol	0 (0.0%)	0 (0.0%)	0 (0.0%)	0 (0.0%)	0 (0.0%)	0 (0.0%)
Height (cm)						
Mean (SD)	159 (6.0)	161 (6.3)	163 (6.3)	163 (6.4)	164 (6.3)	163 (6.4)
Range	132–181	134–182	130–192	110–201	117–198	110–201
Missing	13,896	103	1583	254	1692	17,528
Height (tertiles)						
Shortest tertile	4740 (18.6%)	2935 (39.2%)	4867 (30.3%)	6875 (37.2%)	18,394 (32.8%)	37,811 (30.6%)
Middle tertile	3911 (15.4%)	2359 (31.5%)	4836 (30.1%)	6005 (32.5%)	17,993 (32.1%)	35,104 (28.4%)
Tallest tertile	2883 (11.3%)	2085 (27.9%)	4773 (29.7%)	5350 (28.9%)	17,992 (32.1%)	33,083 (26.8%)
Missing	378 (1.5%)	103 (1.4%)	1583 (9.9%)	254 (1.4%)	1692 (3.0%)	4010 (3.2%)
Missing by protocol	13,518 (53.2%)	0 (0.0%)	0 (0.0%)	0 (0.0%)	0 (0.0%)	13,518 (10.9%)
Diabetes						
No	24,738 (97.3%)	7131 (95.3%)	14,993 (93.4%)	15,827 (85.6%)	45,613 (81.3%)	108,302 (87.7%)
Yes	630 (2.5%)	201 (2.7%)	543 (3.4%)	1038 (5.6%)	4714 (8.4%)	7126 (5.8%)
Missing	62 (0.2%)	150 (2.0%)	523 (3.3%)	1619 (8.8%)	5744 (10.2%)	8098 (6.6%)
Missing by protocol	0 (0.0%)	0 (0.0%)	0 (0.0%)	0 (0.0%)	0 (0.0%)	0 (0.0%)
Colorectal cancer						
Colorectal cancer	519 (2.0%)	127 (1.7%)	151 (0.9%)	92 (0.5%)	97 (0.2%)	986 (0.8%)
No colorectal cancer	24,911 (98.0%)	7355 (98.3%)	15,908 (99.1%)	18,392 (99.5%)	55,974 (99.8%)	122,540 (99.2%)
Cohort						
ATH1	0 (0.0%)	0 (0.0%)	0 (0.0%)	9258 (50.1%)	0 (0.0%)	9258 (7.5%)
ATH2	0 (0.0%)	0 (0.0%)	0 (0.0%)	0 (0.0%)	53,177 (94.8%)	53,177 (43.0%)
FMCF	9631 (37.9%)	0 (0.0%)	0 (0.0%)	0 (0.0%)	0 (0.0%)	9631 (7.8%)
FINRISK	11,912 (46.8%)	7482 (100.0%)	6813 (42.4%)	7571 (41.0%)	2894 (5.2%)	36,672 (29.7%)
H2000	0 (0.0%)	0 (0.0%)	4654 (29.0%)	0 (0.0%)	0 (0.0%)	4654 (3.8%)
HBCS	0 (0.0%)	0 (0.0%)	1071 (6.7%)	0 (0.0%)	0 (0.0%)	1071 (0.9%)
HHS	0 (0.0%)	0 (0.0%)	3521 (21.9%)	1655 (9.0%)	0 (0.0%)	5176 (4.2%)
MFH	3887 (15.3%)	0 (0.0%)	0 (0.0%)	0 (0.0%)	0 (0.0%)	3887 (3.1%)

### 4.1 Relative Risk of CRC in Males and in Females

Former or current smoking was associated with an increased risk of CRC in males (Table 2). The age‐standardized incidence rates of CRC in males were 29.1/10^5^ in never smokers (reference group), 34.8/10^5^ in former smokers (multivariate: HR 1.27, 95% confidence interval (CI) 1.08–1.49), and 39.7/10^5^ in current smokers (multivariate: HR 1.40, 95% CI 1.18–1.66). Males living with overweight or obesity had an increased CRC risk compared with males with normal body weight. The age‐standardized incidence rates of CRC were 29.9/10^5^ in males with normal body weight (reference group), 36.5/10^5^ in males living with overweight (multivariate: HR 1.25, 95% CI 1.09–1.45), and 43.0/10^5^ in males living with obesity (multivariate: HR 1.42, 95% CI 1.17–1.72). In males, the CRC risk was increased in both the middle and tallest height tertiles compared with the shortest tertile. The age‐standardized incidence rates of CRC in males were 27.4/10^5^ in the shortest height tertile (reference group), 35.6/10^5^ in the middle tertile (multivariate: HR 1.21, 95% CI 1.01–1.45), and 39.0/10^5^ in the tallest tertile (multivariate: HR 1.41, 95% CI 1.17–1.70). DM was associated with an increased CRC risk in males. The age‐standardized incidence rates of CRC were 33.7/10^5^ in males who were not diagnosed with DM (reference group) and 40.3/10^5^ in males living with DM (multivariate: HR 1.36, 95% CI 1.04–1.78). Education level, alcohol consumption, or physical inactivity were not statistically significantly associated with the risk of CRC in males in this study (Table 2).

**TABLE 2:** Number of person‐years and incident colorectal cancers, age‐standardized rates per 100,000, and hazard ratios (HR) with 95% confidence intervals (95% CI) in males and females by risk factors.

Males								
Risk factor	Value	Person‐years	Colorectal cancers	Age‐standardized rate	Univariate HR	95% CI	Multivariate HR^a^	95% CI^a^
Education	Low	537,732	398	33.2	Reference, 1.00		Reference, 1.00	
	Middle	401,056	342	34.7	1.15	0.99–1.33	1.13	0.97–1.32
	High	277,444	263	37.5	1.16	0.99–1.36	1.17	0.99–1.37
	Missing	20,598	17	20.3	0.72	0.44–1.18	0.74	0.45–1.22
Smoking	Never smokers	354,604	246	29.1	Reference, 1.00		Reference, 1.00	
	Former smokers	365,296	401	34.8	1.30	1.11–1.53	1.27	1.08–1.49
	Current smokers	508,141	363	39.7	1.38	1.17–1.63	1.40	1.18–1.66
	Missing	8789	10	45.7	0.98	0.52–1.86	1.08	0.55–2.09
Alcohol	No use	114,031	121	37.6	Reference, 1.00		Reference, 1.00	
	Moderate use	528,793	438	33.4	1.04	0.85–1.28	0.99	0.80–1.22
	Heavy use	115,652	91	40.4	1.27	0.96–1.67	1.11	0.83–1.47
	Missing	24,679	21	36.1	0.99	0.62–1.58	1.05	0.64–1.72
	Structurally missing	453,675	349	33.4	1.05	0.85–1.31	1.18	0.89–1.57
Physical activity	Active	185,225	107	30.0	Reference, 1.00		Reference, 1.00	
	Moderately active	448,822	393	32.6	1.12	0.91–1.39	1.11	0.89–1.37
	Inactive	227,882	198	37.7	1.23	0.97–1.56	1.19	0.93–1.51
	Missing	19,433	19	38.7	0.99	0.61–1.62	1.06	0.63–1.78
	Structurally missing	355,468	303	38.0	1.25	0.99–1.58	1.67	1.22–2.27
BMI	Normal weight	523,101	320	29.9	Reference, 1.00		Reference, 1.00	
	Overweight	528,966	508	36.5	1.24	1.07–1.42	1.25	1.09–1.45
	Obesity	149,689	171	43.0	1.40	1.16–1.69	1.42	1.17–1.72
	Missing	35,074	21	21.8	1.10	0.70–1.71	0.00	0.00–Inf
Height	Shortest tertile	338,303	239	27.4	Reference, 1.00		Reference, 1.00	
	Middle tertile	273,800	230	35.6	1.22	1.01–1.46	1.21	1.01–1.45
	Tallest tertile	235,004	227	39.0	1.43	1.19–1.71	1.41	1.17–1.70
	Missing	34,255	21	22.9	1.18	0.76–1.85	—	—
	Structurally missing	355,468	303	38.0	1.30	1.09–1.56	1.00	1.00–1.00
DM	No diabetes	1,179,593	931	33.7	Reference, 1.00		Reference, 1.00	
	Diabetes	33,926	64	40.3	—	—	1.36	1.04–1.78
	Missing	23,311	25	57.5	—	—	1.03	0.68–1.58

Females								
Risk factor	Value	Person‐years	Colorectal cancers	Age‐standardized rate	Univariate HR	95% CI	Multivariate HR^a^	95% CI^a^
Education	Low	639,489	390	26.0	Reference, 1.00		Reference, 1.00	
	Middle	485,791	336	30.3	1.09	0.94–1.26	1.09	0.94–1.27
	High	338,564	242	26.6	1.09	0.93–1.29	1.09	0.92–1.29
	Missing	28,001	18	15.0	0.68	0.42–1.09	0.68	0.42–1.10
Smoking	Never smokers	964,168	717	25.6	Reference, 1.00		Reference, 1.00	
	Former smokers	208,442	100	32.1	0.88	0.71–1.09	0.84	0.67–1.04
	Current smokers	304,026	154	31.2	1.12	0.93–1.34	1.09	0.90–1.31
	Missing	15,210	15	31.9	1.06	0.63–1.78	1.18	0.69–2.01
Alcohol	No use	309,226	270	22.5	Reference, 1.00		Reference, 1.00	
	Moderate use	548,855	314	29.1	1.12	0.94–1.33	1.13	0.94–1.36
	Heavy use	76,565	52	45.3	1.64	1.20–2.25	1.61	1.16–2.24
	Missing	24,946	19	15.0	1.05	0.65–1.69	1.15	0.70–1.88
	Structurally missing	532,254	331	26.4	1.01	0.85–1.21	1.01	0.78–1.29
Physical activity	Active	150,405	81	35.4	Reference, 1.00		Reference, 1.00	
	Moderately active	569,749	341	26.0	0.87	0.68–1.11	0.85	0.66–1.08
	Inactive	353,434	266	29.4	1.02	0.80–1.32	0.99	0.77–1.28
	Missing	23,422	13	9.8	0.54	0.30–0.98	0.52	0.28–0.96
	Structurally missing	394,836	285	24.6	0.94	0.73–1.23	1.04	0.74–1.45
BMI	Normal weight	743,518	363	26.2	Reference, 1.00		Reference, 1.00	
	Overweight	468,293	357	27.1	1.12	0.97–1.30	1.13	0.97–1.32
	Obesity	239,662	240	27.9	1.37	1.16–1.62	1.39	1.17–1.65
	Missing	40,373	26	38.0	1.18	0.79–1.77	0.60	0.08–4.29
Height	Shortest tertile	400,708	227	22.3	Reference, 1.00		Reference, 1.00	
	Middle tertile	352,563	236	28.4	1.18	0.99–1.42	1.19	0.99–1.43
	Tallest tertile	305,404	213	34.2	1.24	1.03–1.49	1.24	1.03–1.50
	Missing	38,336	25	40.6	1.24	0.82–1.88	2.60	0.35–19.35
	Structurally missing	394,836	285	24.6	1.14	0.94–1.38	1.00	1.00–1.00
DM	No diabetes	1,421,522	905	27.0	Reference, 1.00		Reference, 1.00	
	Diabetes	34,921	53	33.7	—	—	1.43	1.07–1.92
	Missing	35,403	28	26.9	—	—	0.95	0.64–1.42

^a^ Adjusted for age, calendar period, education level, cigarette smoking, alcohol consumption, physical activity, BMI, height, and DM.

Heavy alcohol consumption was associated with an increased CRC risk in females (Table 2). The age‐standardized incidence rates of CRC were 22.5/10^5^ in females with no alcohol consumption (reference group), 29.1/10^5^ with moderate consumption (multivariate: HR 1.13, 95% CI 0.94–1.36), and 45.3/10^5^ with heavy alcohol consumption (multivariate: HR 1.61, 95% CI 1.16–2.24). The CRC risk was increased in females living with obesity. The age‐standardized incidence rates of CRC were 26.2/10^5^ in females with normal body weight (reference group), 27.1/10^5^ in females living with overweight (multivariate: HR 1.13, 95% CI 0.97–1.32), and 27.9/10^5^ in females living with obesity (multivariate: HR 1.39, 95% CI 1.17–1.65). Regarding female height, the CRC risk was increased only in the tallest tertile. The age‐standardized incidence rates of CRC in females were 22.3/10^5^ in the shortest height tertile (reference group), 28.4/10^5^ in the middle tertile (multivariate: HR 1.19, 95% CI 0.99–1.43), and 34.2/10^5^ in the tallest tertile (multivariate: HR 1.24, 95% CI 1.03–1.50). DM was associated with an increased CRC risk in females. The age‐standardized incidence rates of CRC were 27.0/10^5^ in females who were not diagnosed with DM (reference group) and 33.7/10^5^ in females living with DM (multivariate: HR 1.43, 95% CI 1.07–1.92). Education level, smoking, or physical inactivity were not statistically significantly associated with the CRC risk in females in this study (Table 2).

### 4.2 CRC Risk Trends

The overall AAPC in CRC incidence adjusted for the considered risk factors was 1.7 (95% CI 0.9–2.6) in males and 1.0 (95% CI 0.1–1.9) in females (Figure 1). Figure 1 illustrates that the AAPC in CRC incidence was positive in almost all risk factor exposure groups but not always statistically significant.

**FIGURE 1:**
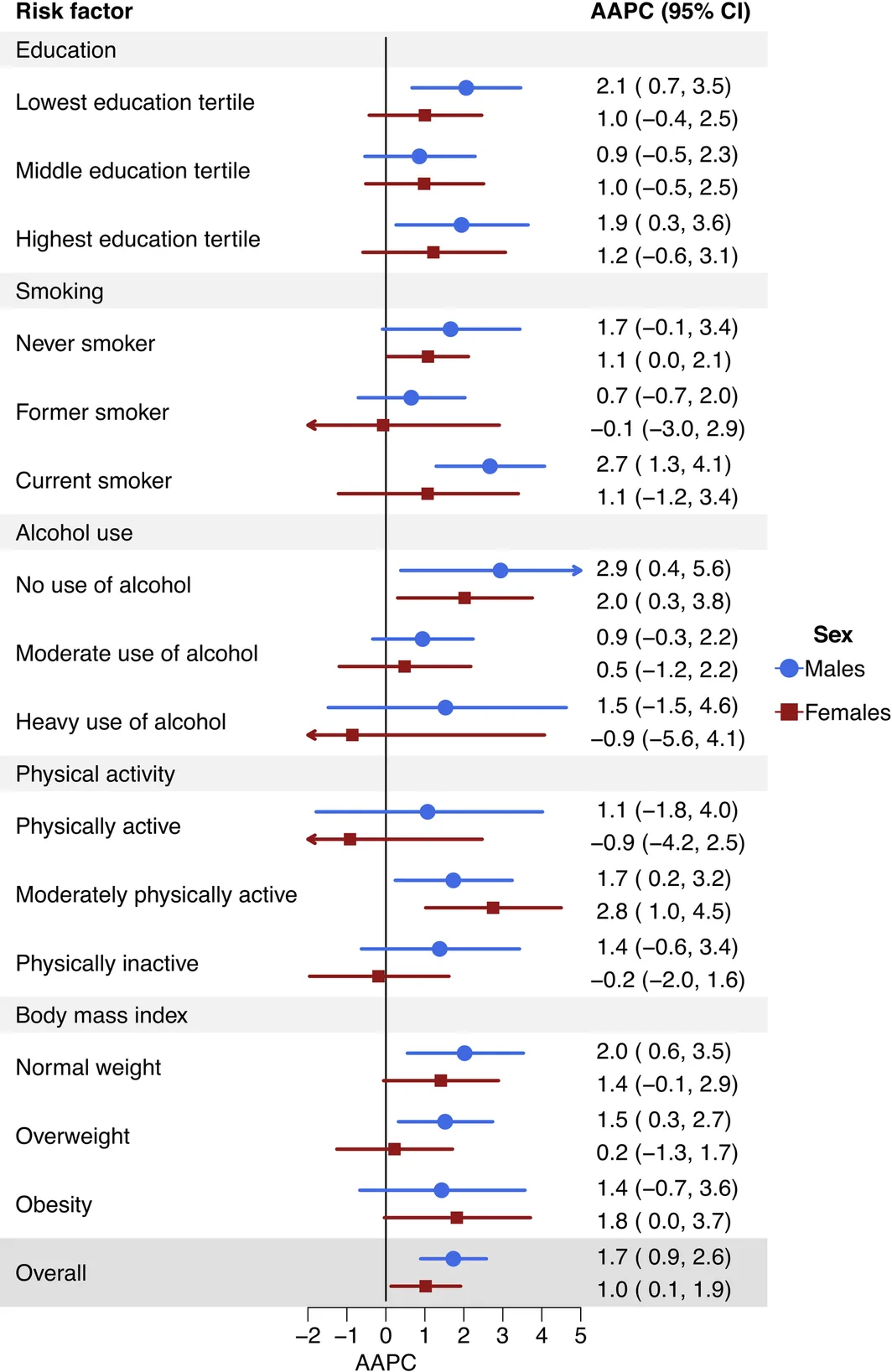
Average annual percentage changes (AAPCs) in colorectal cancer (CRC) incidence adjusted for education, smoking, alcohol consumption, physical activity, body mass index, height, and diabetes (point estimates and 95% confidence interval [95% CI]) by sex and risk factor exposure level.

In males, we found the strongest evidence of increasing incidence in males with low education levels and in current male smokers. When comparing incidence rates by male education level, the AAPC was 2.1% in low (95% CI 0.7–3.5) and 1.9% in high education levels (95% CI 0.3–3.6), without noted trends in males with middle education levels (Figure 1, Table S2). When lifestyle factors, height, and DM are not adjusted, the incidence rate of CRC has risen by 1.7% per year in males with low education levels (95% CI 0.6–2.9) and 1.2% in males with middle education levels (95% CI 0.2–2.2), while no statistically significant trend was observed in males with high education levels (Table S2). In current male smokers, the CRC incidence rate rose 2.7% per year (95% CI 1.3–4.1), while such increases were not noted in male never or former smokers (Figure 1, Table S2).

In females, we found the strongest evidence for increasing incidence in moderately active females. The incidence of CRC rose 2.8% per year in females with moderate physical activity (95% CI 1.0–4.5), without trends in CRC incidences in physically active or inactive females (Figure 1, Table S2).

### 4.3 Sensitivity Analysis

Restricting the sensitivity analysis to 10‐ and 15‐year follow‐up times did not change the conclusions concerning CRC risk trends (Table S3). The shorter the follow‐up time, the more pronounced the effect of current smoking on the CRC trend.

## 5 Discussion

In this study, living with overweight or obesity, having a tall body height, or living with DM were statistically significant risk factors for CRC in both males and females. Furthermore, current or former smoking increased the risk of CRC in males, whereas heavy alcohol consumption increased the CRC risk in females. CRC incidence, adjusted for major lifestyle factors, increased annually by 1.7% in males and by 1.0% in females. In the smoking‐stratified analysis, the average annual percentage increase in the incidence of CRC was especially high in current male smokers. In females, the increase in CRC incidence was especially high in those engaging in moderate physical activity.

### 5.1 Risk Factors

Previous systematic reviews and meta‐analyses [7, 10, 13, 17] along with the International Agency for Research on Cancer [18] have observed the association between smoking and an increased incidence of CRC. Like a previous cohort study from Korea, we noted statistical significance only in males [43].

A meta‐analysis conducted by the World Cancer Research Fund [11], along with other previous systematic reviews and meta‐analyses [7, 10, 13, 17, 19], observed a positive association between alcohol intake and CRC risk. In our study, this was statistically significant only in females with heavy alcohol consumption, while the observed HR was higher (1.61) than previously reported [11]. Compared with previous analyses, the HR in males was of similar magnitude, but did not reach statistical significance [11]. Our non‐user (reference) group included both never and former alcohol users, potentially leading to more pronounced in‐group heterogeneity in males.

Our observation regarding the association between high BMI and an increased risk of CRC agrees with previous findings. Such associations have previously been observed in systematic reviews and meta‐analyses [7, 10, 13, 14], and it has been acknowledged by the International Agency for Research on Cancer [15]. The association between obesity and increased CRC incidence is likely to be influenced by several confounding variables, since many of the factors that cause obesity, such as sedentary behavior and dietary choices, are also known risk factors for CRC [11, 13, 19, 21, 27].

In our study, DM was associated with an increased risk of CRC in both males and females, which has been confirmed in previous systematic reviews and meta‐analyses [8, 9]. The results also strengthen the evidence of an association between greater body height and the risk of CRC. A meta‐analysis conducted by the World Cancer Research Fund [11] observed a 5% increase in the CRC risk for every 5‐cm increase in height, while another meta‐analysis [12] observed a 14% increase in the risk of CRC for every 10‐cm increase in height. Our study found a 24% increase in the risk for females and a 41% increase for males when comparing the tallest height tertile to the shortest tertile. The underlying mechanisms behind the association between taller individual heights and increased CRC incidences may include genetic, environmental, hormonal, and nutritional factors behind greater adult height, including larger numbers of cells and higher concentrations of insulin‐like growth factors [11, 44, 45].

### 5.2 Risk Trends

Although the overall incidence trend of CRC, and the trends of many risk factors for CRC are well studied, there is limited previous research on CRC incidence trends by risk factor status. Overtime changes in substances included in cigarettes, changes in physical activity assessment, and changes in risk behavior in the population may have contributed to the various incidence trends of CRC. According to a 2014 report, the prevalences of various risk factors for CRC show a decreasing trend in the Finnish population, while some, like obesity and alcohol consumption, have become more prevalent [24]. Alcohol consumption has increased and the prevalence of abstinence decreased since the early 1980s until the early 2000s in the Finnish population and has thereafter remained unchanged [24].

A previous Finnish study found that colon cancer incidence was higher in those with higher education levels and higher SES compared with those with lower education levels and lower SES [5]. In our study, education level did not statistically significantly influence the CRC risk or its incidence trends when adjusted for lifestyle factors. If adjustment for lifestyle factors was not performed, the incidence rate of CRC increased especially rapidly in males with low education levels. This suggests that lifestyle factors may be one explanation behind the difference between the incidence trends of CRC at different education levels. Smoking, for example, remains more common in less educated groups, and differences in smoking between educational groups have widened further since the late 1970s [46].

The overall CRC incidence trend has increased especially rapidly in male smokers. They likely suffer from other adverse health issues such as higher consumption of red and processed meats and correspondingly lesser intake of fibers, fruits, and vegetables [47]. While occasional and never smokers improved their dietary behaviors between 1999 and 2013, daily smokers did not, which increased the differences in dietary behaviors between daily and never smokers over time [47]. Notably, in Norway, inactivity appeared more common in male and female smokers compared with their never‐smoker peers [47]. The latency period after smoking initiation for a notable increase in CRC risk is 30–40 years [48]. The prevalence of smoking has decreased in Finnish males since the late 1970s [24, 46].

The amount of leisure‐time physical activity increased in the Finnish population since the late 1970s until the mid‐1990s, when physically active ways of traveling to work became less common, and has thereafter remained somewhat unchanged [24]. In our study, the increase in the incidence of CRC was especially high in moderately active females. Leisure‐time physical activity did not affect the CRC risk in our study. Since half of the females in our study were classified as moderately active, in‐group heterogeneity in physical activity habits remained high, whereas the groups of physically inactive and active females became more strictly defined.

### 5.3 Methodological Considerations

Weaknesses in our study include potential changes in risk factor exposure after the time of survey. This may cause measurement bias leading to misclassification of the exposure. In addition, individuals may underestimate their exposures, which can lead to social desirability bias. Because of these biases, our hazard ratios could be biased toward the null. In our previous sensitivity analyses for the time dependence of exposure effects, measurement bias had no notable effects between exposures and the CRC risk [32]. Further, the validity of our results may have been affected by health survey‐related response biases, since more healthy or more health‐conscious individuals more frequently participate in surveys [49, 50]. Moreover, compared with invited populations, males appear under‐represented in health survey responders [49]. Lower response rates in more recent cohorts compared with older ones may add to selection bias [32]. Adjustment for risk factors remains incomplete to an extent, since some included health studies did not provide information on alcohol, physical activity, or body height. Furthermore, we only adjusted for age, sex, social and behavioral factors, body height, and DM, while adjustments were not made, for example, for certain dietary (e.g., consumption of red meat, lack of dietary fiber), genetic (e.g., family history), or clinical (e.g., inflammatory bowel disease) risk factors. For example, the consumption of red and processed meat has increased in Finland in the past decades [5]. The incompleteness of the adjustment for risk factors should be accounted for when interpreting our results. We do not consider the possibility of chance findings due to multiple comparisons to be a notable problem here, as we mainly report risk factor‐specific trends, and the risk factors in the analysis were carefully chosen according to previous knowledge regarding their associations with CRC risk.

The large numbers of test subjects and the reasonable number of CRCs in our pooled data provided us with good statistical power for the risk factor analysis. The information used on CRC in the analysis was based on extensive and accurate registry data. Data completeness for rectal and anal cancers in the Finnish Cancer Registry was 97.4%. The proportions of morphologically verified colon cancers and rectal and rectosigmoid cancers were 95.8% and 97.1%, respectively [42]. Considering the long latency of CRC, our long follow‐up time was vital. Our results are likely generalizable overall to white, Western populations. Given the adequate representation of the Finnish population provided by the METCA project, we consider the METCA data valid. For example, the large FINRISK survey has, since 1972, been based on random population samples from various parts of Finland drawn from the National Population Information System every 5 years [35]. The participation rates in the FINRISK Study have gradually declined from 91% in males and 94% in females in 1972 to 57% in males and 67% in females in 2012, which can still be considered good response rates.

## 6 Conclusion

Even after adjustment for the major risk factors, the overall incidence of CRC appeared to continue to increase. In risk factor‐stratified analysis, the average annual percentage increase in CRC incidence was especially high in current male smokers. We further confirmed the associations of BMI, body height, and DM with elevated CRC risk. Our findings highlight a need for effective public health strategies, with special emphasis on the prevention of overweight, obesity, and smoking along with other modifiable lifestyle factors known to affect CRC risk.

## Author Contributions

**Emilia Muhonen:** writing – original draft, conceptualization, investigation, writing – review and editing. **Karri Seppä:** methodology, writing – review and editing, investigation, formal analysis, software, data curation, validation. **Heidi Ryynänen:** investigation, formal analysis, software, data curation, validation, writing – review and editing. **Johan G. Eriksson:** investigation, writing – review and editing. **Tommi Härkänen:** investigation, writing – review and editing. **Pekka Jousilahti:** investigation, writing – review and editing. **Paul Knekt:** investigation, writing – review and editing. **Seppo Koskinen:** investigation, writing – review and editing. **Satu Männistö:** investigation, writing – review and editing. **Ossi Rahkonen:** writing – review and editing, investigation. **Maarit A. Laaksonen:** investigation, writing – review and editing. **Janne Pitkäniemi:** conceptualization, investigation, writing – review and editing, funding acquisition, project administration, supervision, methodology, validation. **Sanna Lappi‐Heikkinen:** conceptualization, investigation, writing – review and editing, project administration, supervision, validation.

## Funding

This work was supported by Syöpäsäätiö, Cancer Institute NSW, 2019/CDF1022, LähiTapiola.

## Ethics Statement

The study was approved by the Finnish Institute for Health and Welfare (permits THL/1091/6.02.00/2015 and THL/679/6.02.00/2018). The approval included evaluation of informed consents in each participating cohort. Cancer data were obtained according to Finnish national legislation on the secondary use of health and social data (Act on the Secondary Use of Health and Social Data, 552/2019).

## Conflicts of Interest

The authors declare no conflicts of interest.

## Supporting information


**Table S1:** Height tertiles (cm) by sex and birth cohort.
**Table S2:** Number of incident colorectal cancers (N) per year, age‐standardized rates per 100,000, hazard ratios (HR) for incidence trends and average annual percentage changes (AAPCs) with 95% confidence interval (95% CI) by sex in five time periods from 1972 to 2015.
**Table S3:** Sensitivity analysis for period analysis restricting to 10‐ or 15‐year follow‐up times—number of incident colorectal cancers (N) per year, age‐standardized rates per 100,000, hazard ratios (HR) for incidence trends and average annual percentage changes (AAPCs) with 95% confidence interval (95% CI) by sex in five time periods from 1972 to 2015.

## Data Availability

Permission to use underlying administrative health data from various register keepers for this study can be obtained from Findata, the Finnish Social and Health Data Permit Authority. Requests to access these data can be submitted to Findata at https://findata.fi/en/. Further information is available from the corresponding author upon request.

## References

[1] F. Bray , M. Laversanne , H. Sung , et al., “Global Cancer Statistics 2022: GLOBOCAN Estimates of Incidence and Mortality Worldwide for 36 Cancers in 185 Countries,” CA: A Cancer Journal for Clinicians 74, no. 3 (2024): 229–263, 10.3322/caac.21834. 38572751

[2] R. Sharma , M. Abbasi‐Kangevari , R. Abd‐Rabu , et al., “Global, Regional, and National Burden of Colorectal Cancer and Its Risk Factors, 1990–2019: A Systematic Analysis for the Global Burden of Disease Study 2019,” Lancet Gastroenterology & Hepatology 7, no. 7 (2022): 627–647, 10.1016/S2468-1253(22)00044-9. PMC9192760 35397795

[3] M. Arnold , M. S. Sierra , M. Laversanne , I. Soerjomataram , A. Jemal , and F. Bray , “Global Patterns and Trends in Colorectal Cancer Incidence and Mortality,” Gut 66, no. 4 (2017): 683–691, 10.1136/gutjnl-2015-310912. 26818619

[4] F. Jalili , M. Hajizadeh , S. Mehrabani , S. M. Ghoreishy , and F. MacIsaac , “The Association Between Neighborhood Socioeconomic Status and the Risk of Incidence and Mortality of Colorectal Cancer: A Systematic Review and Meta‐Analysis of 1,678,582 Participants,” Cancer Epidemiology 91 (2024): 102598, 10.1016/j.canep.2024.102598. 38878681

[5] S. Savijärvi , K. Seppä , N. Malila , J. Pitkäniemi , and S. Heikkinen , “Trends of Colorectal Cancer Incidence by Education and Socioeconomic Status in Finland,” Acta Oncologica 58, no. 11 (2019): 1557–1563, 10.1080/0284186X.2019.1652340. 31437070

[6] IARC Working Group on the Evaluation of Cancer‐Preventive Interventions , “Risk Factors and Protective Factors,” in IARC Working Group on the Evaluation of Cancer‐Preventive Interventions. Colorectal Cancer Screening. IARC Handbooks of Cancer Prevention, vol. 17, ed. 50 (International Agency for Research on Cancer, World Health Organization, 2019), 41.

[7] H. Hua , Q. Jiang , P. Sun , and X. Xu , “Risk Factors for Early‐Onset Colorectal Cancer: Systematic Review and Meta‐Analysis,” Frontiers in Oncology 13 (2023): 1132306, 10.3389/fonc.2023.1132306. PMC10196487 37213277

[8] L. Deng , Z. Gui , L. Zhao , J. Wang , and L. Shen , “Diabetes Mellitus and the Incidence of Colorectal Cancer: An Updated Systematic Review and Meta‐Analysis,” Digestive Diseases and Sciences 57, no. 6 (2012): 1576–1585, 10.1007/s10620-012-2055-1. 22350783

[9] H. U. Krämer , B. Schöttker , E. Raum , and H. Brenner , “Type 2 Diabetes Mellitus and Colorectal Cancer: Meta‐Analysis on Sex‐Specific Differences,” European Journal of Cancer 48, no. 9 (2012): 1269–1282, 10.1016/j.ejca.2011.07.010. 21889332

[10] E. Mertens , M. Keuchkarian , M. S. Vasquez , S. Vandevijvere , and J. L. Peñalvo , “Lifestyle Predictors of Colorectal Cancer in European Populations: A Systematic Review,” BMJ Nutrition, Prevention & Health 7, no. 1 (2024): 183–190, 10.1136/bmjnph-2022-000554. PMC11221299 38966096

[11] World Cancer Research Fund/American Institute for Cancer Research , Diet, Nutrition, Physical Activity and Colorectal Cancer: A Global Perspective. Continuous Update Project Expert Report 2018 (World Cancer Research Fund, 2018), 15–84.

[12] E. Zhou , L. Wang , C. N. Santiago , et al., “Adult‐Attained Height and Colorectal Cancer Risk: A Cohort Study, Systematic Review, and Meta‐Analysis,” Cancer Epidemiology, Biomarkers & Prevention 31, no. 4 (2022): 783–792, 10.1158/1055-9965.EPI-21-0398. PMC8983463 35247904

[13] M. Puzzono , A. Mannucci , S. Grannò , et al., “The Role of Diet and Lifestyle in Early‐Onset Colorectal Cancer: A Systematic Review,” Cancers 13, no. 23 (2021): 5933, 10.3390/cancers13235933. PMC8657307 34885046

[14] Z. Ungvari , M. Fekete , P. Varga , et al., “Overweight and Obesity Significantly Increase Colorectal Cancer Risk: A Meta‐Analysis of 66 Studies Revealing a 25–57% Elevation in Risk,” GeroScience 47, no. 3 (2025): 3343–3364, 10.1007/s11357-024-01375-x. PMC12181496 39379738

[15] IARC Working Group on the Evaluation of Cancer‐Preventive Interventions , “Cancer‐Preventive Effects in Humans,” in IARC Working Group on the Evaluation of Cancer‐Preventive Interventions. Absence of Excess Body Fatness. IARC Handbooks of Cancer Prevention, vol. 16 (International Agency for Research on Cancer, World Health Organization, 2021), 74–112. 37200467

[16] X. Diao , Y. Ling , Y. Zeng , et al., “Physical Activity and Cancer Risk: A Dose‐Response Analysis for the Global Burden of Disease Study 2019,” Cancer Communications 43, no. 11 (2023): 1229–1243, 10.1002/cac2.12488. PMC10631483 37743572

[17] J. Wieser , M. Hoffmeister , H. Brenner , and U. Mons , “Associations of Alcohol Use and Smoking With Early‐Onset Colorectal Cancer—A Systematic Review and Meta‐Analysis,” Clinical Colorectal Cancer 24, no. 3 (2025): 331–340.e15, 10.1016/j.clcc.2025.05.002. 40544033

[18] IARC Working Group on the Evaluation of Carcinogenic Risk to Humans , “Tobacco Smoking,” in IARC Working Group on the Evaluation of Carcinogenic Risk to Humans. Personal Habits and Indoor Combustions. IARC Monographs on the Evaluation of Carcinogenic Risks to Humans, vol. 100E, ed. 87 (International Agency for Research on Cancer, 2012), 80. PMC4781577 23193840

[19] A. R. Vieira , L. Abar , D. S. M. Chan , et al., “Foods and Beverages and Colorectal Cancer Risk: A Systematic Review and Meta‐Analysis of Cohort Studies, an Update of the Evidence of the WCRF‐AICR Continuous Update Project,” Annals of Oncology 28, no. 8 (2017): 1788–1802, 10.1093/annonc/mdx171. 28407090

[20] Z. Ungvari , M. Fekete , P. Varga , et al., “Association Between Red and Processed Meat Consumption and Colorectal Cancer Risk: A Comprehensive Meta‐Analysis of Prospective Studies,” GeroScience 47, no. 3 (2025): 5123–5140, 10.1007/s11357-025-01646-1. PMC12181564 40210826

[21] IARC Monographs Working Group , “Cancer of the Colorectum,” in IARC Monographs Working Group. Red Meat and Processed Meat. IARC Monographs on the Evaluation of Carcinogenic Risks to Humans, vol. 114 (International Agency for Research on Cancer, 2018), 111–247.

[22] N. Papadimitriou , G. Markozannes , A. Kanellopoulou , et al., “An Umbrella Review of the Evidence Associating Diet and Cancer Risk at 11 Anatomical Sites,” Nature Communications 12, no. 1 (2021): 4579, 10.1038/s41467-021-24861-8. PMC8319326 34321471

[23] N. Keum , D. Aune , D. C. Greenwood , W. Ju , and E. L. Giovannucci , “Calcium Intake and Colorectal Cancer Risk: Dose‐Response Meta‐Analysis of Prospective Observational Studies: Calcium Intake and Colorectal Cancer,” International Journal of Cancer 135, no. 8 (2014): 1940–1948, 10.1002/ijc.28840. 24623471

[24] A. Helldán and S. Helakorpi , “Health Behaviour and Health Among the Finnish Adult Population, Spring 2014 [Suomalaisen Aikuisväestön Terveyskäyttäytyminen Ja Terveys, Kevät 2014],” Helsinki: THL Finnish Institute for Health and Welfare (2015): 12–21.

[25] N. E. Kaartinen , H. Tapanainen , S. Männistö , et al., “Changes in Food Consumption and Nutrient Intake in Finnish Adults 1997–2017: The National FinDiet Survey [Aikuisväestön Ruoankäytön ja Ravintoaineiden Saannin Muutokset Vuosina 1997–2017: Kansallinen FinRavinto‐Tutkimus],” Lääkärilehti 76 (2021): 273–278.

[26] L. Valsta , N. Kaartinen , H. Tapanainen , S. Männistö , and K. Sääksjärvi , “Nutrition in Finland—The National FinDiet 2017 Survey [Ravitsemus Suomessa – FinRavinto 2017 ‐Tutkimus],” Helsinki: Finnish Institute for Health and Welfare (THL) (2018): 49–134.

[27] S. P. Mambrini , F. Menichetti , S. Ravella , et al., “ Ultra‐Processed Food Consumption and Incidence of Obesity and Cardiometabolic Risk Factors in Adults: A Systematic Review of Prospective Studies,” Nutrients 15, no. 11 (2023): 2583, 10.3390/nu15112583. PMC10255607 37299546

[28] R. L. Bhardwaj , A. Parashar , H. P. Parewa , and L. Vyas , “An Alarming Decline in the Nutritional Quality of Foods: The Biggest Challenge for Future Generations' Health,” Food 13, no. 6 (2024): 877, 10.3390/foods13060877. PMC10969708 38540869

[29] M. N. Woessner , A. Tacey , A. Levinger‐Limor , A. G. Parker , P. Levinger , and I. Levinger , “The Evolution of Technology and Physical Inactivity: The Good, the Bad, and the Way Forward,” Frontiers in Public Health 9 (2021): 655491, 10.3389/fpubh.2021.655491. PMC8193221 34123989

[30] D. Hoffmann , M. V. Djordjevic , and K. D. Brunnemann , “Changes in Cigarette Design and Composition Over Time and How They Influence the Yields of Smoke Constituents,” in National Institutes of Health, National Cancer Institute. The FTC Cigarette Test Method for Determining Tar, Nicotine, and Carbon Monoxide Yields of U.S. Cigarettes. Report of the NCI Expert Committee. Smoking and Tobacco Control Monograph, vol. 7 (U.S. Department of Health and Human Services, Public Health Service, National Institutes of Health, 1996), 15–37.

[31] P. Mäkelä and K. Warpenius , “Alcohol Consumption, Drinking Habits and Alcohol‐Related Harm in Finland. Results from the Drinking Habits Survey [Suomalaisten alkoholinkäyttö, juomatavat ja alkoholihaitat: Juomatapatutkimuksen tuloksia],” Helsinki: Finnish Institute for Health and Welfare THL (2024): 38–44.

[32] J. Pitkäniemi , S. Heikkinen , K. Seppä , et al., “Pooling of Finnish Population‐Based Health Studies: Lifestyle Risk Factors of Colorectal and Lung Cancer,” Acta Oncologica 59, no. 11 (2020): 1338–1342, 10.1080/0284186X.2020.1789214. 32657191

[33] E. Lahelma , A. Aittomäki , M. Laaksonen , et al., “Cohort Profile: The Helsinki Health Study,” International Journal of Epidemiology 42, no. 3 (2013): 722–730, 10.1093/ije/dys039. 22467288

[34] P. Knekt , H. Rissanen , R. Järvinen , and M. Heliövaara , “Cohort Profile: The Finnish Mobile Clinic Health Surveys FMC, FMCF and MFS,” International Journal of Epidemiology 46, no. 6 (2017): 1760–1761i, 10.1093/ije/dyx092. 29040560

[35] K. Borodulin , H. Tolonen , P. Jousilahti , et al., “Cohort Profile: The National FINRISK Study,” International Journal of Epidemiology 47, no. 3 (2018): 696–696i, 10.1093/ije/dyx239. 29165699

[36] S. Heistaro , “Methodology Report Health 2000 Survey,” Helsinki: National Public Health Institute (2008): 1–246.

[37] J. Eriksson , “Early Growth and Coronary Heart Disease and Type 2 Diabetes: Experiences From the Helsinki Birth Cohort Studies,” International Journal of Obesity 30 (2006): 18–22, 10.1038/sj.ijo.0803515. 17133231

[38] T. Härkänen , R. Kaikkonen , E. Virtala , and S. Koskinen , “Inverse Probability Weighting and Doubly Robust Methods in Correcting the Effects of Non‐Response in the Reimbursed Medication and Self‐Reported Turnout Estimates in the ATH Survey,” BMC Public Health 14, no. 1 (2014): 1150, 10.1186/1471-2458-14-1150. PMC4246429 25373328

[39] E. Vartiainen , “Validation of Self Reported Smoking by Serum Cotinine Measurement in a Community‐Based Study,” Journal of Epidemiology and Community Health 56, no. 3 (2002): 167–170, 10.1136/jech.56.3.167. PMC1732104 11854334

[40] J. Rantala , K. Seppä , J. Eriksson , et al., “Incidence Trends of Early‐Onset Breast Cancer by Lifestyle Risk Factors,” BMC Cancer 25, no. 1 (2025): 326, 10.1186/s12885-025-13730-y. PMC11846213 39984883

[41] WHO Consultation on Obesity (1999: Geneva, Switzerland) & World Health Organization , Obesity: Preventing and Managing the Global Epidemic. Report of a WHO Consultation, ed. World Health Organization (World Health Organization, 2000), 6–15. 11234459

[42] M. K. Leinonen , J. Miettinen , S. Heikkinen , J. Pitkäniemi , and N. Malila , “Quality Measures of the Population‐Based Finnish Cancer Registry Indicate Sound Data Quality for Solid Malignant Tumours,” European Journal of Cancer 77 (2017): 31–39, 10.1016/j.ejca.2017.02.017. 28350996

[43] S. Nam , Y. J. Choi , D. W. Kim , E. C. Park , and J. G. Kang , “Risk Factors for Colorectal Cancer in Korea: A Population‐Based Retrospective Cohort Study,” Annals of Coloproctology 35, no. 6 (2019): 347–356, 10.3393/ac.2019.10.21. PMC6968718 31937075

[44] D. Gunnell , M. Okasha , G. Davey Smith , S. E. Oliver , J. Sandhu , and J. M. P. Holly , “Height, Leg Length, and Cancer Risk: A Systematic Review,” Epidemiologic Reviews 23, no. 2 (2001): 313–342, 10.1093/oxfordjournals.epirev.a000809. 12192740

[45] D. Albanes and M. Winick , “Are Cell Number and Cell Proliferation Risk Factors for Cancer?,” Journal of the National Cancer Institute 80, no. 10 (1988): 772–775, 10.1093/jnci/80.10.772. 3385783

[46] O. Ruokolainen , A. Heloma , P. Jousilahti , et al., “Thirty‐Eight‐Year Trends of Educational Differences in Smoking in Finland,” International Journal of Public Health 64, no. 6 (2019): 853–860, 10.1007/s00038-019-01228-x. PMC6614163 30906956

[47] G. Sæbø and E. Kvaavik , “Accumulation of Material and Lifestyle Problems Among Daily Smokers in Norway 1999 to 2013 ‐ a Cross‐Sectional Analysis,” BMC Public Health 16, no. 1 (2016): 781, 10.1186/s12889-016-3465-3. PMC4983036 27519316

[48] H. Kuper , P. Boffetta , and H.‐O. Adami , “Tobacco Use and Cancer Causation: Association by Tumour Type,” Journal of Internal Medicine 252, no. 3 (2002): 206–224, 10.1046/j.1365-2796.2002.01022.x. 12270001

[49] E. Strandhagen , C. Berg , L. Lissner , et al., “Selection Bias in a Population Survey With Registry Linkage: Potential Effect on Socioeconomic Gradient in Cardiovascular Risk,” European Journal of Epidemiology 25, no. 3 (2010): 163–172, 10.1007/s10654-010-9427-7. 20127393

[50] A. J. Søgaard , R. Selmer , E. Bjertness , and D. Thelle , “The Oslo Health Study: The Impact of Self‐Selection in a Large, Population‐Based Survey,” International Journal for Equity in Health 3, no. 1 (2004): 3, 10.1186/1475-9276-3-3. PMC428581 15128460

