# Supplementary material for: Incidence Trends of Colorectal Cancer by Social and Behavioral Factors—A Pooled Cohort Study
Source: Int J Cancer. 2026 Aug 5;159(12):2897–914. doi: 10.1002/ijc.70656 (PMC13688214; doi:10.1002/ijc.70656)
Supplement: Supplementary file 1 [file IJC-159-2897-s001.pdf]

# Supplementary Tables

# Incidence trends of colorectal cancer by social and behavioral factors – a pooled cohort study

Emilia Muhonen, Karri Seppä, Heidi Ryynänen, Johan G Eriksson, Tommi Härkänen, Pekka Jousilahti, Paul Knekt, Seppo Koskinen, Satu Männistö, Ossi Rahkonen, Maarit A Laaksonen, Janne Pitkäniemi, Sanna Lappi-Heikkinen, METCA Study Group

**Contents**

**Supplementary Table S1** Height tertiles (cm) by sex and birth cohort

**Supplementary Table S2** Number of incident colorectal cancers (N) per year, age-standardized rates per 100,000, hazard ratios (HR) for incidence trends and average annual percentage changes (AAPCs) with 95% confidence interval (95% CI) by sex in five time periods from 1972 to 2015

**Supplementary Table S3** Sensitivity analysis for period analysis restricting to 10- or 15-year follow-up times – number of incident colorectal cancers (N) per year, age-standardized rates per 100,000, hazard ratios (HR) for incidence trends and average annual percentage changes (AAPCs) with 95% confidence interval (95% CI) by sex in five time periods from 1972 to 2015

**Supplementary Table S1** Height tertiles (cm) by sex and birth cohort

| | Males | | Females | |
|---|---|---|---|---|
| Birth cohort | First cut-off point | Second cut-off point | First cut-off point | Second cut-off point |
| –1919 | 168 | 173 | 154 | 159 |
| 1920–1929 | 170 | 175 | 156 | 161 |
| 1930–1939 | 171 | 177 | 158 | 163 |
| 1940–1949 | 173 | 178 | 160 | 165 |
| 1950–1959 | 175 | 180 | 161 | 166 |
| 1960–1969 | 176 | 182 | 163 | 168 |
| 1970–1979 | 176 | 182 | 163 | 168 |
| 1980–2016 | 176 | 182 | 163 | 168 |

**Supplementary Table S2** Number of incident colorectal cancers (N) per year, age-standardized rates per 100,000, hazard ratios (HR) for incidence trends and average annual percentage changes (AAPCs) with 95% confidence interval (95% CI) by sex in five time periods from 1972 to 2015

**Education**

| | | Lowest tertile | | | | Middle tertile | | | | Highest tertile | | | |
|---|---|---|---|---|---|---|---|---|---|---|---|---|---|
| Sex | Period | N per year[1] | Age-stand. rate[2] | HR[3] | 95% CI | N per year[1] | Age-stand. rate[2] | HR[3] | 95% CI | N per year[1] | Age-stand. rate[2] | HR[3] | 95% CI |
| males | 1972–1981 | 1.2 | 22.5 | 0.48 | (0.24–0.97) | 1.8 | 26.7 | 0.68 | (0.35–1.31) | 0.3 | 8.9 | 0.14 | (0.04–0.5) |
| | 1982–1991 | 3.7 | 30 | 0.6 | (0.38–0.94) | 4.2 | 28.2 | 0.68 | (0.43–1.09) | 3.1 | 42.3 | 0.69 | (0.4–1.17) |
| | 1992–2001 | 7.8 | 37.9 | 0.75 | (0.53–1.06) | 9 | 49.2 | 1.12 | (0.77–1.63) | 6.2 | 47.5 | 0.94 | (0.62–1.43) |
| | 2002–2011 | 17.7 | 44.3 | 0.98 | (0.75–1.29) | 12.6 | 49.6 | 1.03 | (0.74–1.42) | 10.8 | 49.7 | 1.02 | (0.72–1.44) |
| | 2012–2015 | 23.5 | 44.7 | reference, 1 | | 16.5 | 42 | reference, 1 | | 14.6 | 44.6 | reference, 1 | |
| | AAPC[4] (unadjusted) | - | - | 1.74 | (0.59–2.9) | - | - | 1.19 | (0.17–2.22) | - | - | 0.87 | (-0.42–2.17) |
| | AAPC[4] (adjusted[3]) | - | - | 2.06 | (0.67–3.46) | - | - | 0.86 | (-0.54–2.29) | - | - | 1.94 | (0.26–3.65) |
| females | 1972–1981 | 0.9 | 21.1 | 0.36 | (0.16–0.8) | 1.5 | 23.7 | 0.65 | (0.32–1.31) | 1 | 23.8 | 0.9 | (0.39–2.08) |
| | 1982–1991 | 4.5 | 24.6 | 0.73 | (0.46–1.15) | 6 | 36.5 | 0.9 | (0.57–1.4) | 2.7 | 29.2 | 0.91 | (0.52–1.61) |
| | 1992–2001 | 9.3 | 31.1 | 0.97 | (0.68–1.38) | 7.1 | 29.5 | 0.73 | (0.49–1.08) | 4.6 | 28.1 | 0.92 | (0.59–1.45) |
| | 2002–2011 | 16 | 36.2 | 1.01 | (0.76–1.35) | 12 | 35.1 | 0.84 | (0.61–1.16) | 9.6 | 33.5 | 1.05 | (0.74–1.48) |
| | 2012–2015 | 20.9 | 36.4 | reference, 1 | | 17.5 | 36.5 | reference, 1 | | 15.7 | 34.4 | reference, 1 | |
| | AAPC[4] (unadjusted) | - | - | 1.31 | (0.23–2.41) | - | - | 0.83 | (-0.22–1.9) | - | - | 1.48 | (0.16–2.82) |
| | AAPC[4] (adjusted[3]) | - | - | 1 | (-0.43–2.46) | - | - | 0.98 | (-0.52–2.51) | - | - | 1.22 | (-0.59–3.07) |

[1] Average annual number of colorectal cancers, [2] Adjusted for age, rates are comparable between calendar periods and sex, [3] Adjusted for age and time since study adjusted by age, cigarette smoking, alcohol consumption, physical activity, BMI, height, and DM. [4] Average annual percentage change during 1972–2015.

**Smoking**

| | | Never smokers | | | | Former smokers | | | | Current smokers | | | |
|---|---|---|---|---|---|---|---|---|---|---|---|---|---|
| Sex | Period | N per year[1] | Age-stand. rate[2] | HR[3] | 95% CI | N per year[1] | Age-stand. rate[2] | HR[3] | 95% CI | N per year[1] | Age-stand. rate[2] | HR[3] | 95% CI |
| males | 1972–1981 | 0.7 | 19.6 | 0.5 | (0.2–1.23) | 1 | 20.2 | 0.53 | (0.25–1.12) | 1.6 | 25.7 | 0.34 | (0.17–0.67) |
| | 1982–1991 | 1.6 | 18 | 0.46 | (0.25–0.86) | 4.7 | 36.1 | 0.95 | (0.62–1.46) | 4.7 | 38.4 | 0.5 | (0.32–0.79) |
| | 1992–2001 | 5.2 | 37.1 | 0.88 | (0.58–1.35) | 8.4 | 40.4 | 1.07 | (0.76–1.52) | 9.4 | 53.9 | 0.78 | (0.54–1.13) |
| | 2002–2011 | 10.4 | 39.3 | 0.96 | (0.68–1.34) | 16.3 | 52.6 | 1.17 | (0.89–1.55) | 15.2 | 50.5 | 0.91 | (0.66–1.25) |
| | 2012–2015 | 16.6 | 38.1 | reference, 1 | | 24.2 | 43.2 | reference, 1 | | 13.4 | 52.5 | reference, 1 | |
| | AAPC[4] (unadjusted) | - | - | 1.55 | (0.16–2.97) | - | - | 1.29 | (0.26–2.33) | - | - | 1.38 | (0.29–2.49) |
| | AAPC[4] (adjusted[3]) | - | - | 1.66 | (-0.09–3.44) | - | - | 0.65 | (-0.71–2.03) | - | - | 2.67 | (1.29–4.07) |
| females | 1972–1981 | 3 | 23.1 | 0.58 | (0.35–0.94) | 0.3 | 39.3 | 1.32 | (0.32–5.38) | 0.1 | 2.6 | 0.21 | (0.03–1.71) |
| | 1982–1991 | 11.4 | 30.7 | 0.86 | (0.63–1.18) | 0.8 | 22.3 | 0.91 | (0.35–2.33) | 1.2 | 30.8 | 0.75 | (0.35–1.61) |
| | 1992–2001 | 15.8 | 27.5 | 0.82 | (0.63–1.07) | 1.6 | 23.9 | 0.88 | (0.44–1.74) | 3.6 | 43 | 1.09 | (0.64–1.88) |
| | 2002–2011 | 26.1 | 38.2 | 0.91 | (0.73–1.13) | 4 | 26.3 | 0.92 | (0.56–1.52) | 7.5 | 36 | 1.13 | (0.72–1.75) |
| | 2012–2015 | 38.5 | 37.8 | reference, 1 | | 8.4 | 33.4 | reference, 1 | | 7.4 | 34.6 | reference, 1 | |
| | AAPC[4] (unadjusted) | - | - | 1.04 | (0.29–1.79) | - | - | 0.09 | (-2.13–2.37) | - | - | 1.9 | (0.03–3.79) |
| | AAPC[4] (adjusted[3]) | - | - | 1.08 | (0.04–2.12) | - | - | -0.07 | (-2.95–2.91) | - | - | 1.07 | (-1.22–3.4) |

[1] Average annual number of colorectal cancers, [2]Adjusted for age, rates are comparable between calendar periods and sex, [3] Adjusted for age and time since study adjusted by age, education level, alcohol consumption, physical activity, BMI, height, and DM. [4] Average annual percentage change during 1972–2015.

**Alcohol consumption**

| | | No use | | | | Moderate use | | | | Heavy use | | | |
|---|---|---|---|---|---|---|---|---|---|---|---|---|---|
| Sex | Period | N per year[1] | Age-stand. rate[2] | HR[3] | 95% CI | N per year[1] | Age-stand. rate[2] | HR[3] | 95% CI | N per year[1] | Age-stand. rate[2] | HR[3] | 95% CI |
| males | 1972–1981 | 0.6 | 17.4 | 0.28 | (0.09–0.88) | 1.3 | 25.6 | 0.53 | (0.27–1.03) | 0.1 | 14 | 0.25 | (0.03–2.15) |
| | 1982–1991 | 1.5 | 25.9 | 0.43 | (0.19–0.94) | 3.7 | 33.8 | 0.73 | (0.47–1.13) | 0.6 | 33.4 | 0.6 | (0.21–1.71) |
| | 1992–2001 | 1.9 | 29.2 | 0.55 | (0.28–1.1) | 9.4 | 52.2 | 1.1 | (0.81–1.51) | 2 | 49.9 | 1.23 | (0.62–2.47) |
| | 2002–2011 | 3.7 | 74 | 0.84 | (0.49–1.42) | 18.1 | 43 | 1.1 | (0.85–1.41) | 4 | 65.4 | 1.18 | (0.69–2.03) |
| | 2012–2015 | 11 | 42.9 | reference, 1 | | 28.1 | 39.8 | reference, 1 | | 5.9 | 53.5 | reference, 1 | |
| | AAPC[4] (unadjusted) | - | - | 2.57 | (0.86–4.3) | - | - | 0.3 | (-0.67–1.27) | - | - | 1.54 | (-0.74–3.87) |
| | AAPC[4] (adjusted[3]) | - | - | 2.94 | (0.38–5.56) | - | - | 0.94 | (-0.34–2.24) | - | - | 1.53 | (-1.48–4.63) |
| females | 1972–1981 | 1.9 | 22.6 | 0.48 | (0.24–0.98) | 0.7 | 29.9 | 0.99 | (0.41–2.36) | 0 | 0 | 0 | (0–Inf) |
| | 1982–1991 | 5.6 | 29 | 0.7 | (0.43–1.16) | 1.4 | 24.5 | 0.62 | (0.33–1.16) | 0 | 0 | 0 | (0–Inf) |
| | 1992–2001 | 4.5 | 22.6 | 0.54 | (0.34–0.86) | 5.6 | 36.2 | 1.04 | (0.72–1.51) | 1 | 109.6 | 2.06 | (0.84–5.01) |
| | 2002–2011 | 7.7 | 25.1 | 0.75 | (0.52–1.09) | 13.7 | 33.4 | 0.98 | (0.75–1.29) | 2.4 | 56.9 | 1.21 | (0.64–2.28) |
| | 2012–2015 | 18.1 | 43.4 | reference, 1 | | 25 | 34.6 | reference, 1 | | 4.4 | 34.8 | reference, 1 | |
| | AAPC[4] (unadjusted) | - | - | 0.78 | (-0.31–1.88) | - | - | 0.31 | (-1.01–1.65) | - | - | 0.81 | (-3.08–4.85) |
| | AAPC[4] (adjusted[3]) | - | - | 2.02 | (0.3–3.76) | - | - | 0.48 | (-1.2–2.18) | - | - | -0.86 | (-5.57–4.07) |

[1] Average annual number of colorectal cancers, [2]Adjusted for age, rates are comparable between calendar periods and sex, [3] Adjusted for age and time since study adjusted by age, education level, cigarette smoking, physical activity, BMI, height, and DM. [4] Average annual percentage change during 1972–2015.

**Physical activity**

| | | Active | | | | Moderately active | | | | Inactive | | | |
|---|---|---|---|---|---|---|---|---|---|---|---|---|---|
| Sex | Period | N per year[1] | Age-stand. rate[2] | HR[3] | 95% CI | N per year[1] | Age-stand. rate[2] | HR[3] | 95% CI | N per year[1] | Age-stand. rate[2] | HR[3] | 95% CI |
| males | 1972–1981 | 0.1 | 5.4 | 0.52 | (0.06–4.36) | 0.5 | 7.3 | 0.36 | (0.14–0.95) | 0.7 | 16.3 | 0.72 | (0.27–1.88) |
| | 1982–1991 | 0.5 | 18.3 | 0.56 | (0.2–1.62) | 2.7 | 30.7 | 0.6 | (0.36–0.97) | 2 | 24.5 | 0.68 | (0.36–1.28) |
| | 1992–2001 | 2.4 | 36 | 1.12 | (0.6–2.08) | 7.4 | 41.9 | 0.84 | (0.6–1.17) | 3.4 | 41.6 | 0.73 | (0.44–1.2) |
| | 2002–2011 | 5 | 41.2 | 0.96 | (0.58–1.58) | 16.8 | 45.8 | 0.94 | (0.73–1.22) | 7.9 | 55 | 1.02 | (0.69–1.5) |
| | 2012–2015 | 6.7 | 34.9 | reference, 1 | | 29.6 | 48.5 | reference, 1 | | 14.4 | 45.9 | reference, 1 | |
| | AAPC[4] (unadjusted) | - | - | -0.18 | (-2.69–2.38) | - | - | 1.78 | (0.54–3.03) | - | - | 1.35 | (-0.22–2.95) |
| | AAPC[4] (adjusted[3]) | - | - | 1.07 | (-1.79–4.02) | - | - | 1.73 | (0.24–3.24) | - | - | 1.38 | (-0.63–3.43) |
| females | 1972–1981 | 0 | 0 | 0 | (0–Inf) | 0.3 | 5.2 | 0.24 | (0.07–0.81) | 0.5 | 7.1 | 0.46 | (0.16–1.31) |
| | 1982–1991 | 0.8 | 31.9 | 1.45 | (0.55–3.79) | 2 | 15.9 | 0.53 | (0.3–0.93) | 3.4 | 27.4 | 0.94 | (0.55–1.61) |
| | 1992–2001 | 1.5 | 32.3 | 0.95 | (0.46–1.99) | 5.8 | 31.9 | 0.77 | (0.54–1.12) | 6.1 | 31.4 | 0.91 | (0.6–1.39) |
| | 2002–2011 | 3.3 | 29.7 | 0.82 | (0.47–1.43) | 14.7 | 30.6 | 0.88 | (0.68–1.15) | 9.6 | 42.3 | 0.88 | (0.62–1.24) |
| | 2012–2015 | 6.2 | 38 | reference, 1 | | 28.1 | 36.3 | reference, 1 | | 17.3 | 38.6 | reference, 1 | |
| | AAPC[4] (unadjusted) | - | - | 0.79 | (-1.93–3.6) | - | - | 2.18 | (0.8–3.57) | - | - | 1.24 | (-0.13–2.64) |
| | AAPC[4] (adjusted[3]) | - | - | -0.92 | (-4.2–2.47) | - | - | 2.75 | (1.02–4.5) | - | - | -0.19 | (-1.96–1.62) |

[1] Average annual number of colorectal cancers, [2]Adjusted for age, rates are comparable between calendar periods and sex, [3] Adjusted for age and time since study adjusted by age, education level, cigarette smoking, alcohol consumption, BMI, height, and DM. [4] Average annual percentage change during 1972–2015.

**BMI**

| Sex | Period | Normal weight N per year[1] | Age-stand. rate[2] | HR[3] | 95% CI | Overweight N per year[1] | Age-stand. rate[2] | HR[3] | 95% CI | Obesity N per year[1] | Age-stand. rate[2] | HR[3] | 95% CI |
|---|---|---|---|---|---|---|---|---|---|---|---|---|---|
| males | 1972–1981 | 1.4 | 20.7 | 0.52 | (0.25–1.06) | 1.5 | 21.9 | 0.42 | (0.22–0.79) | 0.4 | 25.6 | 0.37 | (0.11–1.18) |
| | 1982–1991 | 3.1 | 22.7 | 0.58 | (0.35–0.96) | 5.7 | 35.1 | 0.6 | (0.41–0.87) | 2.1 | 48.5 | 0.88 | (0.45–1.72) |
| | 1992–2001 | 6.6 | 33.8 | 0.91 | (0.62–1.36) | 12.6 | 48.3 | 0.84 | (0.63–1.13) | 3.8 | 47.1 | 1.2 | (0.71–2.03) |
| | 2002–2011 | 14.4 | 45.3 | 1.24 | (0.9–1.72) | 19.5 | 43.9 | 0.84 | (0.66–1.09) | 6.9 | 65.9 | 1.21 | (0.79–1.85) |
| | 2012–2015 | 16.2 | 33.1 | reference, 1 | | 28.7 | 50.5 | reference, 1 | | 9.5 | 45.9 | reference, 1 | |
| | AAPC[4] (unadjusted) | - | - | 1.47 | (0.34–2.62) | - | - | 1.14 | (0.2–2.1) | - | - | 1.15 | (-0.38–2.71) |
| | AAPC[4] (adjusted[3]) | - | - | 2.02 | (0.55–3.53) | - | - | 1.52 | (0.32–2.74) | - | - | 1.43 | (-0.67–3.57) |
| females | 1972–1981 | 0.5 | 10.1 | 0.23 | (0.08–0.61) | 1.6 | 31.5 | 0.86 | (0.43–1.7) | 1.1 | 23.6 | 0.64 | (0.28–1.46) |
| | 1982–1991 | 4.1 | 28.3 | 0.76 | (0.48–1.21) | 5.3 | 29.2 | 0.99 | (0.63–1.56) | 3.8 | 30.3 | 0.79 | (0.46–1.37) |
| | 1992–2001 | 8.1 | 27.8 | 0.91 | (0.64–1.29) | 8.3 | 29.2 | 0.98 | (0.67–1.43) | 4.2 | 42.6 | 0.61 | (0.38–0.99) |
| | 2002–2011 | 14.8 | 29.9 | 0.89 | (0.67–1.19) | 12.8 | 29.5 | 0.95 | (0.69–1.29) | 9.5 | 51.5 | 1.02 | (0.71–1.46) |
| | 2012–2015 | 22 | 34.8 | reference, 1 | | 19 | 32 | reference, 1 | | 13.4 | 44.3 | reference, 1 | |
| | AAPC[4] (unadjusted) | - | - | 1.65 | (0.51–2.8) | - | - | 0.5 | (-0.55–1.56) | - | - | 1.82 | (0.53–3.13) |
| | AAPC[4] (adjusted[3]) | - | - | 1.41 | (-0.06–2.89) | - | - | 0.22 | (-1.26–1.71) | - | - | 1.82 | (-0.04–3.71) |

[1] Average annual number of colorectal cancers, [2]Adjusted for age, rates are comparable between calendar periods and sex, [3] Adjusted for age and time since study adjusted by age, education level, cigarette smoking, alcohol consumption, physical activity, height, and DM. [4] Average annual percentage change during 1972–2015.

**Supplementary Table S3** Sensitivity analysis for period analysis restricting to 10- or 15-year follow-up times – number of incident colorectal cancers (N) per year, age-standardized rates per 100,000, hazard ratios (HR) for incidence trends and average annual percentage changes (AAPCs) with 95% confidence interval (95% CI) by sex in five time periods from 1972 to 2015

**Education** (Sensitivity analysis restricting to 10-year follow-up time)

| | | Lowest tertile | | | | Middle tertile | | | | Highest tertile | | | |
|---|---|---|---|---|---|---|---|---|---|---|---|---|---|
| Sex | Period | N per year[1] | Age-stand. rate[2] | HR[3] | 95% CI | N per year[1] | Age-stand. rate[2] | HR[3] | 95% CI | N per year[1] | Age-stand. rate[2] | HR[3] | 95% CI |
| males | 1972–1981 | 1.2 | 22.5 | 0.23 | (0.08–0.68) | 1.8 | 26.7 | 0.64 | (0.19–2.14) | 0.3 | 8.9 | 0.28 | (0.05–1.65) |
| | 1982–1991 | 1.4 | 26.8 | 0.25 | (0.09–0.67) | 1.5 | 23.7 | 0.58 | (0.18–1.83) | 1.6 | 66.6 | 1.43 | (0.38–5.35) |
| | 1992–2001 | 1 | 97.7 | 0.72 | (0.35–1.51) | 1.3 | 98.6 | 2.03 | (1.01–4.07) | 0.9 | 33.9 | 1.08 | (0.5–2.32) |
| | 2002–2011 | 5 | 56.2 | 1.22 | (0.79–1.89) | 4.1 | 74.2 | 1.73 | (1.03–2.89) | 2.3 | 37.3 | 0.8 | (0.47–1.38) |
| | 2012–2015 | 12.8 | 40.2 | reference, 1 | | 7.5 | 36.9 | reference, 1 | | 10.4 | 54.1 | reference, 1 | |
| | AAPC[4] (unadjusted) | - | - | 1.75 | (0.18–3.34) | - | - | 1.39 | (0.05–2.75) | - | - | 0.97 | (-0.82–2.79) |
| | AAPC[4] (adjusted[3]) | - | - | 3.35 | (0.53–6.24) | - | - | -0.34 | (-3.21–2.62) | - | - | 0.77 | (-2.68–4.33) |
| females | 1972–1981 | 0.9 | 21.1 | 0.27 | (0.07–0.98) | 1.5 | 23.7 | 0.36 | (0.09–1.41) | 1 | 23.8 | 1.08 | (0.21–5.51) |
| | 1982–1991 | 1.9 | 25.1 | 0.57 | (0.19–1.74) | 2 | 29.2 | 0.45 | (0.12–1.65) | 1.1 | 31.8 | 1.13 | (0.26–4.86) |
| | 1992–2001 | 1.5 | 41.1 | 1.08 | (0.57–2.07) | 0.7 | 33.5 | 0.8 | (0.34–1.86) | 0.9 | 31.2 | 1.02 | (0.47–2.17) |
| | 2002–2011 | 3.1 | 28.7 | 0.75 | (0.46–1.23) | 2.7 | 30 | 0.74 | (0.43–1.28) | 2.6 | 35.3 | 0.8 | (0.48–1.35) |
| | 2012–2015 | 13.1 | 44.1 | reference, 1 | | 8.8 | 36.4 | reference, 1 | | 10.6 | 42.3 | reference, 1 | |
| | AAPC[4] (unadjusted) | - | - | 1.93 | (0.39–3.49) | - | - | 1.15 | (-0.28–2.6) | - | - | 1.19 | (-0.48–2.89) |
| | AAPC[4] (adjusted[3]) | - | - | 1.24 | (-1.77–4.35) | - | - | 2.86 | (-0.56–6.4) | - | - | 0.53 | (-2.91–4.1) |

[1] Average annual number of colorectal cancers, [2]Adjusted for age, rates are comparable between calendar periods and sex, [3] Adjusted for age and time since study adjusted by age, cigarette smoking, alcohol consumption, physical activity, BMI, height, and DM. [4] Average annual percentage change during 1972–2015.

**Smoking** (Sensitivity analysis restricting to 10-year follow-up time)

| | | Never smokers | | | | Former smokers | | | | Current smokers | | | |
|---|---|---|---|---|---|---|---|---|---|---|---|---|---|
| Sex | Period | N per year[1] | Age-stand. rate[2] | HR[3] | 95% CI | N per year[1] | Age-stand. rate[2] | HR[3] | 95% CI | N per year[1] | Age-stand. rate[2] | HR[3] | 95% CI |
| males | 1972–1981 | 0.7 | 19.6 | 0.56 | (0.07–4.67) | 1 | 20.2 | 0.47 | (0.16–1.4) | 1.6 | 25.7 | 0.16 | (0.05–0.51) |
| | 1982–1991 | 0.5 | 15.4 | 0.43 | (0.06–3.15) | 1.9 | 28.3 | 0.72 | (0.29–1.76) | 2.1 | 58.4 | 0.24 | (0.08–0.72) |
| | 1992–2001 | 0.8 | 31.8 | 1.13 | (0.5–2.55) | 1.7 | 76.2 | 1.28 | (0.72–2.28) | 0.8 | 182.4 | 0.8 | (0.34–1.9) |
| | 2002–2011 | 2.9 | 39.7 | 1.22 | (0.73–2.04) | 6.3 | 72.4 | 1.42 | (0.97–2.09) | 2.4 | 44.2 | 0.74 | (0.38–1.44) |
| | 2012–2015 | 9.6 | 36.1 | reference, 1 | | 16.2 | 43.4 | reference, 1 | | 5.1 | 47.9 | reference, 1 | |
| | AAPC[4] (unadjusted) | - | - | 2.13 | (0.18–4.11) | - | - | 1.57 | (0.23–2.93) | - | - | 1.14 | (-0.39–2.7) |
| | AAPC[4] (adjusted[3]) | - | - | 0.3 | (-3.44–4.19) | - | - | 0.14 | (-2.28–2.61) | - | - | 4.37 | (1.29–7.55) |
| females | 1972–1981 | 3 | 23.1 | 0.55 | (0.2–1.5) | 0.3 | 39.3 | 1.01 | (0.15–6.99) | 0.1 | 2.6 | 0.05 | (0–0.74) |
| | 1982–1991 | 3.9 | 26 | 0.73 | (0.29–1.82) | 0.6 | 35.1 | 1 | (0.2–4.93) | 0.5 | 80.4 | 0.23 | (0.03–1.7) |
| | 1992–2001 | 1.9 | 34.8 | 0.86 | (0.51–1.46) | 0.4 | 26.2 | 0.96 | (0.31–2.97) | 0.8 | 35.2 | 1.32 | (0.52–3.34) |
| | 2002–2011 | 5.4 | 34.4 | 0.73 | (0.51–1.04) | 1.3 | 22.7 | 0.72 | (0.34–1.54) | 1.4 | 19.7 | 0.7 | (0.31–1.57) |
| | 2012–2015 | 24.7 | 43 | reference, 1 | | 5.5 | 33.8 | reference, 1 | | 3 | 36.7 | reference, 1 | |
| | AAPC[4] (unadjusted) | - | - | 1.54 | (0.52–2.56) | - | - | -0.76 | (-3.46–2.01) | - | - | 2.23 | (-0.6–5.13) |
| | AAPC[4] (adjusted[3]) | - | - | 1.49 | (-0.8–3.84) | - | - | 0.3 | (-4.12–4.92) | - | - | 1.91 | (-2.98–7.06) |

[1] Average annual number of colorectal cancers, [2]Adjusted for age, rates are comparable between calendar periods and sex, [3] Adjusted for age and time since study adjusted by age, education level, alcohol consumption, physical activity, BMI, height, and DM. [4] Average annual percentage change during 1972–2015.

**Alcohol** (Sensitivity analysis restricting to 10-year follow-up time)

| | | No use | | | | Moderate use | | | | Heavy use | | | |
|---|---|---|---|---|---|---|---|---|---|---|---|---|---|
| Sex | Period | N per year[1] | Age-stand. rate[2] | HR[3] | 95% CI | N per year[1] | Age-stand. rate[2] | HR[3] | 95% CI | N per year[1] | Age-stand. rate[2] | HR[3] | 95% CI |
| males | 1972–1981 | 0.6 | 17.4 | 0 | (0–Inf) | 1.3 | 25.6 | 0 | (0–Inf) | 0.1 | 14 | 0 | (0–Inf) |
| | 1982–1991 | 0.7 | 30.1 | 0 | (0–Inf) | 1.5 | 35.4 | 0 | (0–Inf) | 0.3 | 61 | 0 | (0–Inf) |
| | 1992–2001 | 0 | 0 | 0 | (0–Inf) | 2.6 | 101.7 | 1.29 | (0.8–2.09) | 0.5 | 210.8 | 1.69 | (0.56–5.04) |
| | 2002–2011 | 1.1 | 61.9 | 0.97 | (0.48–1.96) | 6.9 | 50.3 | 1.3 | (0.92–1.84) | 1.7 | 120 | 1.44 | (0.68–3.06) |
| | 2012–2015 | 8.9 | 43.6 | reference, 1 | | 18.3 | 39.2 | reference, 1 | | 3.4 | 65.3 | reference, 1 | |
| | AAPC[4] (unadjusted) | - | - | 2.83 | (0.74–4.97) | - | - | 0.56 | (-0.69–1.83) | - | - | 1.59 | (-1.4–4.67) |
| | AAPC[4] (adjusted[3]) | - | - | 6.41 | (-1.17–14.57) | - | - | -0.29 | (-2.68–2.15) | - | - | -1.88 | (-7.37–3.94) |
| females | 1972–1981 | 1.9 | 22.6 | 0 | (0–Inf) | 0.7 | 29.9 | 0.9 | (0.08–10.04) | 0 | 0 | 0 | (0–Inf) |
| | 1982–1991 | 2.1 | 27.1 | 0 | (0–Inf) | 0.4 | 20 | 0.4 | (0.06–2.97) | 0 | 0 | 0 | (0–Inf) |
| | 1992–2001 | 0.5 | 32.9 | 0.74 | (0.28–1.94) | 2.1 | 28 | 1.09 | (0.64–1.86) | 0.2 | 16.2 | 0.56 | (0.12–2.56) |
| | 2002–2011 | 2.1 | 26.8 | 0.82 | (0.48–1.38) | 4.6 | 27.2 | 0.74 | (0.49–1.11) | 0.9 | 34.2 | 0.58 | (0.24–1.4) |
| | 2012–2015 | 13.6 | 37.7 | reference, 1 | | 15.8 | 38 | reference, 1 | | 3.2 | 43.6 | reference, 1 | |
| | AAPC[4] (unadjusted) | - | - | 0.88 | (-0.45–2.23) | - | - | 0.8 | (-1.04–2.67) | - | - | 4.69 | (-2.3–12.17) |
| | AAPC[4] (adjusted[3]) | - | - | 1.77 | (-2.28–5.99) | - | - | 0.69 | (-2.27–3.74) | - | - | 2.78 | (-4.9–11.09) |

[1] Average annual number of colorectal cancers, [2]Adjusted for age, rates are comparable between calendar periods and sex, [3] Adjusted for age and time since study adjusted by age, education level, cigarette smoking, physical activity, BMI, height, and DM. [4] Average annual percentage change during 1972–2015.

**Physical activity** (Sensitivity analysis restricting to 10-year follow-up time)

| | | Active | | | | Moderately active | | | | Inactive | | | |
|---|---|---|---|---|---|---|---|---|---|---|---|---|---|
| Sex | Period | N per year[1] | Age-stand. rate[2] | HR[3] | 95% CI | N per year[1] | Age-stand. rate[2] | HR[3] | 95% CI | N per year[1] | Age-stand. rate[2] | HR[3] | 95% CI |
| males | 1972–1981 | 0.1 | 5.4 | 1.76 | (0.14–22.15) | 0.5 | 7.3 | 0.25 | (0.08–0.79) | 0.7 | 16.3 | 0.45 | (0.12–1.73) |
| | 1982–1991 | 0.4 | 37.2 | 2.76 | (0.56–13.67) | 1 | 15.8 | 0.39 | (0.16–0.94) | 0.6 | 21.7 | 0.25 | (0.07–0.94) |
| | 1992–2001 | 0.7 | 40.5 | 2.16 | (0.78–6) | 1.7 | 37.6 | 0.92 | (0.52–1.63) | 0.8 | 87.6 | 1.1 | (0.48–2.51) |
| | 2002–2011 | 1.1 | 26.8 | 0.89 | (0.37–2.13) | 7.1 | 59.1 | 1.19 | (0.82–1.71) | 2.8 | 65 | 1.19 | (0.7–2.02) |
| | 2012–2015 | 3.1 | 37.8 | reference, 1 | | 17.7 | 44.4 | reference, 1 | | 10.6 | 47.3 | reference, 1 | |
| | AAPC[4] (unadjusted) | - | - | -0.63 | (-4.16–3.02) | - | - | 2.17 | (0.43–3.93) | - | - | 0.73 | (-1.37–2.88) |
| | AAPC[4] (adjusted[3]) | - | - | -2.52 | (-7.08–2.26) | - | - | 2.63 | (0.27–5.06) | - | - | 0.47 | (-3.07–4.13) |
| females | 1972–1981 | 0 | 0 | 0 | (0–Inf) | 0.3 | 5.2 | 0.21 | (0.05–0.88) | 0.5 | 7.1 | 0.17 | (0.03–0.81) |
| | 1982–1991 | 0.4 | 30.2 | 3 | (0.5–18.21) | 0.9 | 13.2 | 0.43 | (0.16–1.18) | 1 | 18.2 | 0.31 | (0.08–1.2) |
| | 1992–2001 | 0.3 | 21.3 | 0.91 | (0.24–3.45) | 2 | 48.5 | 1.03 | (0.6–1.76) | 0.8 | 28.8 | 0.77 | (0.34–1.73) |
| | 2002–2011 | 0.4 | 8.7 | 0.3 | (0.09–0.95) | 5.6 | 29.8 | 0.75 | (0.51–1.11) | 2.2 | 37.8 | 0.74 | (0.43–1.28) |
| | 2012–2015 | 3.3 | 37.5 | reference, 1 | | 18.9 | 41.2 | reference, 1 | | 12.3 | 43.8 | reference, 1 | |
| | AAPC[4] (unadjusted) | - | - | -0.01 | (-4.12–4.28) | - | - | 2.54 | (0.59–4.52) | - | - | 2.26 | (0.15–4.42) |
| | AAPC[4] (adjusted[3]) | - | - | -2.31 | (-8.12–3.86) | - | - | 2.58 | (-0.06–5.3) | - | - | 2.12 | (-1.54–5.9) |

[1] Average annual number of colorectal cancers, [2]Adjusted for age, rates are comparable between calendar periods and sex, [3] Adjusted for age and time since study adjusted by age, education level, cigarette smoking, alcohol consumption, BMI, height, and DM. [4] Average annual percentage change during 1972–2015.

**BMI** (Sensitivity analysis restricting to 10-year follow-up time)

| | | Normal weight | | | | Overweight | | | | Obesity | | | |
|---|---|---|---|---|---|---|---|---|---|---|---|---|---|
| Sex | Period | N per year[1] | Age-stand. rate[2] | HR[3] | 95% CI | N per year[1] | Age-stand. rate[2] | HR[3] | 95% CI | N per year[1] | Age-stand. rate[2] | HR[3] | 95% CI |
| males | 1972–1981 | 1.4 | 20.7 | 0.26 | (0.07–0.89) | 1.5 | 21.9 | 0.48 | (0.16–1.4) | 0.4 | 25.6 | 0.39 | (0.08–1.76) |
| | 1982–1991 | 1.4 | 25.8 | 0.3 | (0.09–0.97) | 2 | 36 | 0.59 | (0.22–1.54) | 1.1 | 48.5 | 0.8 | (0.25–2.57) |
| | 1992–2001 | 0.5 | 17.6 | 0.67 | (0.25–1.79) | 1.7 | 99.3 | 1.08 | (0.6–1.92) | 1 | 92.8 | 1.59 | (0.72–3.53) |
| | 2002–2011 | 3.3 | 61.6 | 1.36 | (0.81–2.29) | 4.5 | 42.7 | 0.92 | (0.61–1.39) | 3.4 | 82.1 | 1.52 | (0.87–2.69) |
| | 2012–2015 | 9.5 | 36.7 | reference, 1 | | 15.7 | 48.4 | reference, 1 | | 6.8 | 49 | reference, 1 | |
| | AAPC[4] (unadjusted) | - | - | 1.69 | (0.17–3.23) | - | - | 1.23 | (-0.09–2.56) | - | - | 1.18 | (-0.74–3.12) |
| | AAPC[4] (adjusted[3]) | - | - | 3.59 | (0.22–7.07) | - | - | 0.32 | (-2.19–2.89) | - | - | 1.14 | (-2.16–4.55) |
| females | 1972–1981 | 0.5 | 10.1 | 0.2 | (0.04–0.89) | 1.6 | 31.5 | 0.69 | (0.18–2.56) | 1.1 | 23.6 | 0.28 | (0.06–1.27) |
| | 1982–1991 | 2.2 | 37.5 | 0.82 | (0.25–2.76) | 1.3 | 15.9 | 0.5 | (0.14–1.75) | 1.4 | 25.5 | 0.37 | (0.09–1.51) |
| | 1992–2001 | 1.1 | 25.9 | 0.92 | (0.45–1.85) | 1.3 | 55.8 | 1.52 | (0.77–2.99) | 0.7 | 23 | 0.76 | (0.32–1.79) |
| | 2002–2011 | 2.6 | 24.9 | 0.61 | (0.36–1.02) | 3.1 | 28.3 | 0.93 | (0.57–1.53) | 2.2 | 40.6 | 0.7 | (0.39–1.24) |
| | 2012–2015 | 12.8 | 41.1 | reference, 1 | | 11.8 | 35.3 | reference, 1 | | 9 | 46.6 | reference, 1 | |
| | AAPC[4] (unadjusted) | - | - | 1.78 | (0.23–3.36) | - | - | 1.31 | (-0.14–2.78) | - | - | 1.32 | (-0.34–3.01) |
| | AAPC[4] (adjusted[3]) | - | - | 2.4 | (-0.87–5.78) | - | - | 0.17 | (-2.96–3.39) | - | - | 2.56 | (-1.1–6.36) |

[1] Average annual number of colorectal cancers, [2]Adjusted for age, rates are comparable between calendar periods and sex, [3] Adjusted for age and time since study adjusted by age, education level, cigarette smoking, alcohol consumption, physical activity, height, and DM. [4] Average annual percentage change during 1972–2015.

**Education** (Sensitivity analysis restricting to 15-year follow-up time)

| | | Lowest tertile | | | | Middle tertile | | | | Highest tertile | | | |
|---|---|---|---|---|---|---|---|---|---|---|---|---|---|
| Sex | Period | N per year[1] | Age-stand. rate[2] | HR[3] | 95% CI | N per year[1] | Age-stand. rate[2] | HR[3] | 95% CI | N per year[1] | Age-stand. rate[2] | HR[3] | 95% CI |
| males | 1972–1981 | 1.2 | 22.5 | 0.35 | (0.14–0.86) | 1.8 | 26.7 | 0.63 | (0.27–1.44) | 0.3 | 8.9 | 0.43 | (0.09–1.98) |
| | 1982–1991 | 3.2 | 31.2 | 0.44 | (0.21–0.9) | 3.6 | 30.1 | 0.63 | (0.3–1.31) | 2.7 | 49.6 | 1.75 | (0.62–4.94) |
| | 1992–2001 | 2.1 | 67.5 | 0.63 | (0.35–1.13) | 2.6 | 68.8 | 1.56 | (0.89–2.74) | 1.2 | 27.8 | 1.06 | (0.53–2.12) |
| | 2002–2011 | 5.9 | 51 | 1.07 | (0.73–1.57) | 4.9 | 68 | 1.31 | (0.85–2.02) | 3.4 | 39.7 | 1.01 | (0.63–1.61) |
| | 2012–2015 | 15.6 | 46.6 | reference, 1 | | 10.9 | 42.7 | reference, 1 | | 10.8 | 46.5 | reference, 1 | |
| | AAPC[4] (unadjusted) | - | - | 1.72 | (0.37–3.08) | - | - | 1.4 | (0.25–2.56) | - | - | 0.83 | (-0.72–2.42) |
| | AAPC[4] (adjusted[3]) | - | - | 2.95 | (0.58–5.37) | - | - | 0.95 | (-1.39–3.34) | - | - | 0.18 | (-2.84–3.3) |
| females | 1972–1981 | 0.9 | 21.1 | 0.22 | (0.08–0.55) | 1.5 | 23.7 | 0.38 | (0.15–0.96) | 1 | 23.8 | 0.99 | (0.32–3.03) |
| | 1982–1991 | 3.1 | 21.8 | 0.35 | (0.17–0.72) | 4.1 | 30 | 0.5 | (0.23–1.1) | 2.4 | 33.2 | 1.06 | (0.41–2.74) |
| | 1992–2001 | 3.1 | 42.1 | 0.98 | (0.58–1.65) | 1.7 | 33 | 0.71 | (0.37–1.36) | 1.6 | 32.1 | 1.21 | (0.65–2.28) |
| | 2002–2011 | 4.5 | 31.4 | 0.78 | (0.52–1.16) | 3.3 | 29 | 0.63 | (0.4–0.99) | 3.6 | 36.8 | 0.95 | (0.61–1.49) |
| | 2012–2015 | 17.1 | 43 | reference, 1 | | 13.4 | 39 | reference, 1 | | 12.2 | 37.4 | reference, 1 | |
| | AAPC[4] (unadjusted) | - | - | 1.95 | (0.66–3.26) | - | - | 1.21 | (0–2.44) | - | - | 0.98 | (-0.44–2.42) |
| | AAPC[4] (adjusted[3]) | - | - | 2.28 | (-0.16–4.78) | - | - | 3.03 | (0.42–5.72) | - | - | 0.44 | (-2.43–3.39) |

[1] Average annual number of colorectal cancers, [2]Adjusted for age, rates are comparable between calendar periods and sex, [3] Adjusted for age and time since study adjusted by age, cigarette smoking, alcohol consumption, physical activity, BMI, height, and DM. [4] Average annual percentage change during 1972–2015.

**Smoking** (Sensitivity analysis restricting to 15-year follow-up time)

| | | Never smokers | | | | Former smokers | | | | Current smokers | | | |
|---|---|---|---|---|---|---|---|---|---|---|---|---|---|
| Sex | Period | N per year[1] | Age-stand. rate[2] | HR[3] | 95% CI | N per year[1] | Age-stand. rate[2] | HR[3] | 95% CI | N per year[1] | Age-stand. rate[2] | HR[3] | 95% CI |
| males | 1972–1981 | 0.7 | 19.6 | 0.97 | (0.25–3.8) | 1 | 20.2 | 0.4 | (0.16–0.96) | 1.6 | 25.7 | 0.36 | (0.14–0.9) |
| | 1982–1991 | 1.4 | 19.7 | 0.96 | (0.3–3.11) | 4.5 | 41.2 | 0.79 | (0.42–1.5) | 3.6 | 42.9 | 0.42 | (0.18–0.94) |
| | 1992–2001 | 1.1 | 28 | 0.97 | (0.47–1.97) | 3 | 56.3 | 1.07 | (0.66–1.72) | 1.8 | 128.5 | 0.8 | (0.4–1.6) |
| | 2002–2011 | 3.8 | 39.5 | 1.17 | (0.75–1.84) | 7.4 | 65.2 | 1.19 | (0.85–1.67) | 3.2 | 42.3 | 0.94 | (0.55–1.61) |
| | 2012–2015 | 11.4 | 37.6 | reference, 1 | | 19.9 | 46.4 | reference, 1 | | 6.3 | 44 | reference, 1 | |
| | AAPC[4] (unadjusted) | - | - | 2.2 | (0.51–3.92) | - | - | 1.1 | (-0.02–2.23) | - | - | 1.28 | (-0.09–2.68) |
| | AAPC[4] (adjusted[3]) | - | - | 0.52 | (-2.63–3.77) | - | - | 0.88 | (-1.18–2.98) | - | - | 3.41 | (0.78–6.12) |
| females | 1972–1981 | 3 | 23.1 | 0.42 | (0.22–0.81) | 0.3 | 39.3 | 1.88 | (0.37–9.59) | 0.1 | 2.6 | 0.07 | (0.01–0.6) |
| | 1982–1991 | 8.1 | 27 | 0.56 | (0.32–0.98) | 0.8 | 25.7 | 1.37 | (0.4–4.7) | 0.9 | 36.7 | 0.25 | (0.08–0.77) |
| | 1992–2001 | 4.2 | 32.1 | 0.89 | (0.58–1.35) | 0.8 | 26.8 | 1.11 | (0.47–2.66) | 1.4 | 37.8 | 0.98 | (0.46–2.11) |
| | 2002–2011 | 7 | 34.4 | 0.74 | (0.54–1) | 1.7 | 22.6 | 0.77 | (0.41–1.44) | 2.4 | 26.3 | 0.77 | (0.43–1.38) |
| | 2012–2015 | 30.1 | 43.1 | reference, 1 | | 7.1 | 33.7 | reference, 1 | | 5.8 | 43.9 | reference, 1 | |
| | AAPC[4] (unadjusted) | - | - | 1.33 | (0.47–2.2) | - | - | -0.39 | (-2.76–2.04) | - | - | 2.53 | (0.31–4.8) |
| | AAPC[4] (adjusted[3]) | - | - | 2.03 | (0.2–3.89) | - | - | -1.41 | (-5.13–2.46) | - | - | 4.47 | (0.75–8.33) |

[1] Average annual number of colorectal cancers, [2]Adjusted for age, rates are comparable between calendar periods and sex, [3] Adjusted for age and time since study adjusted by age, education level, alcohol consumption, physical activity, BMI, height, and DM. [4] Average annual percentage change during 1972–2015.

**Alcohol** (Sensitivity analysis restricting to 15-year follow-up time)

| | | No use | | | | Moderate use | | | | Heavy use | | | |
|---|---|---|---|---|---|---|---|---|---|---|---|---|---|
| Sex | Period | N per year[1] | Age-stand. rate[2] | HR[3] | 95% CI | N per year[1] | Age-stand. rate[2] | HR[3] | 95% CI | N per year[1] | Age-stand. rate[2] | HR[3] | 95% CI |
| males | 1972–1981 | 0.6 | 17.4 | 0.17 | (0.01–2.58) | 1.3 | 25.6 | 0.35 | (0.13–0.92) | 0.1 | 14 | 0.73 | (0.04–14.87) |
| | 1982–1991 | 1.4 | 28.9 | 0.26 | (0.02–3.59) | 3.4 | 38.1 | 0.5 | (0.22–1.16) | 0.5 | 37.3 | 1.45 | (0.15–13.97) |
| | 1992–2001 | 0.2 | 20.2 | 0.3 | (0.04–2.25) | 3.8 | 66.4 | 1.17 | (0.76–1.79) | 0.5 | 92.4 | 1.12 | (0.39–3.21) |
| | 2002–2011 | 1.3 | 68.2 | 0.99 | (0.51–1.9) | 8.6 | 45.2 | 1.17 | (0.86–1.6) | 2.3 | 109 | 1.35 | (0.72–2.56) |
| | 2012–2015 | 9.4 | 43.4 | reference, 1 | | 21.7 | 39.6 | reference, 1 | | 4.7 | 59.4 | reference, 1 | |
| | AAPC[4] (unadjusted) | - | - | 2.7 | (0.84–4.59) | - | - | 0.26 | (-0.82–1.36) | - | - | 2.57 | (-0.22–5.44) |
| | AAPC[4] (adjusted[3]) | - | - | 4.42 | (-1.33–10.5) | - | - | 0.17 | (-1.98–2.36) | - | - | -0.72 | (-5.68–4.51) |
| females | 1972–1981 | 1.9 | 22.6 | 0.37 | (0.09–1.48) | 0.7 | 29.9 | 0.92 | (0.22–3.76) | 0 | 0 | 0.16 | (0–Inf) |
| | 1982–1991 | 4.2 | 25.2 | 0.44 | (0.12–1.65) | 0.9 | 23.5 | 0.47 | (0.14–1.64) | 0 | 0 | 0 | (0–Inf) |
| | 1992–2001 | 0.8 | 21.7 | 0.44 | (0.17–1.15) | 2.7 | 36.3 | 1.1 | (0.69–1.76) | 0.5 | 46.3 | 1.34 | (0.48–3.77) |
| | 2002–2011 | 2.3 | 23.5 | 0.7 | (0.43–1.15) | 6.4 | 29.3 | 0.84 | (0.6–1.18) | 1.8 | 52.2 | 1.01 | (0.52–1.98) |
| | 2012–2015 | 15.4 | 41.2 | reference, 1 | | 20.5 | 36.6 | reference, 1 | | 4.4 | 40.7 | reference, 1 | |
| | AAPC[4] (unadjusted) | - | - | 0.92 | (-0.29–2.15) | - | - | 0.83 | (-0.76–2.43) | - | - | 2.32 | (-2.43–7.3) |
| | AAPC[4] (adjusted[3]) | - | - | 2.94 | (-0.51–6.52) | - | - | 0.21 | (-2.36–2.85) | - | - | -0.62 | (-6.23–5.33) |

[1] Average annual number of colorectal cancers, [2]Adjusted for age, rates are comparable between calendar periods and sex, [3] Adjusted for age and time since study adjusted by age, education level, cigarette smoking, physical activity, BMI, height, and DM. [4] Average annual percentage change during 1972–2015.

**Physical activity** (Sensitivity analysis restricting to 15-year follow-up time)

| | | Active | | | | Moderately active | | | | Inactive | | | |
|---|---|---|---|---|---|---|---|---|---|---|---|---|---|
| Sex | Period | N per year[1] | Age-stand. rate[2] | HR[3] | 95% CI | N per year[1] | Age-stand. rate[2] | HR[3] | 95% CI | N per year[1] | Age-stand. rate[2] | HR[3] | 95% CI |
| males | 1972–1981 | 0.1 | 5.4 | 1.89 | (0.18–20.14) | 0.5 | 7.3 | 0.31 | (0.11–0.88) | 0.7 | 16.3 | 0.82 | (0.26–2.63) |
| | 1982–1991 | 0.5 | 24.7 | 2.28 | (0.56–9.22) | 1.9 | 41.7 | 0.45 | (0.23–0.88) | 1.8 | 30.1 | 0.72 | (0.28–1.85) |
| | 1992–2001 | 0.9 | 32.1 | 1.8 | (0.75–4.33) | 2.9 | 38.7 | 0.9 | (0.57–1.43) | 1.4 | 79.1 | 0.91 | (0.45–1.88) |
| | 2002–2011 | 1.4 | 24 | 0.78 | (0.38–1.63) | 8.9 | 55 | 1.13 | (0.82–1.55) | 3.4 | 63.8 | 1.22 | (0.76–1.98) |
| | 2012–2015 | 4.4 | 37.7 | reference, 1 | | 21.9 | 46.9 | reference, 1 | | 11.2 | 46 | reference, 1 | |
| | AAPC[4] (unadjusted) | - | - | 0.24 | (-2.97–3.57) | - | - | 1.94 | (0.47–3.42) | - | - | 0.61 | (-1.17–2.41) |
| | AAPC[4] (adjusted[3]) | - | - | -2.07 | (-6.23–2.27) | - | - | 2.36 | (0.32–4.45) | - | - | 0.37 | (-2.64–3.49) |
| females | 1972–1981 | 0 | 0 | 0 | (0–Inf) | 0.3 | 5.2 | 0.19 | (0.05–0.68) | 0.5 | 7.1 | 0.17 | (0.05–0.56) |
| | 1982–1991 | 0.8 | 36.5 | 1.44 | (0.4–5.17) | 1.7 | 15.7 | 0.39 | (0.19–0.8) | 2 | 18 | 0.31 | (0.13–0.74) |
| | 1992–2001 | 0.7 | 35.1 | 1.23 | (0.47–3.17) | 2.8 | 34.1 | 0.82 | (0.52–1.31) | 2.4 | 46.6 | 0.95 | (0.51–1.76) |
| | 2002–2011 | 1 | 27.4 | 0.52 | (0.24–1.16) | 7.3 | 30.3 | 0.75 | (0.54–1.03) | 2.8 | 38.7 | 0.76 | (0.47–1.23) |
| | 2012–2015 | 4.6 | 38.5 | reference, 1 | | 25.5 | 40.4 | reference, 1 | | 13.5 | 43.8 | reference, 1 | |
| | AAPC[4] (unadjusted) | - | - | -0.82 | (-3.69–2.14) | - | - | 2.29 | (0.71–3.9) | - | - | 2.14 | (0.45–3.85) |
| | AAPC[4] (adjusted[3]) | - | - | -1.46 | (-5.57–2.82) | - | - | 3.44 | (1.23–5.7) | - | - | 2.57 | (-0.42–5.65) |

[1] Average annual number of colorectal cancers, [2]Adjusted for age, rates are comparable between calendar periods and sex, [3] Adjusted for age and time since study adjusted by age, education level, cigarette smoking, alcohol consumption, BMI, height, and DM. [4] Average annual percentage change during 1972–2015.

**BMI** (Sensitivity analysis restricting to 15-year follow-up time)

| | | Normal weight | | | | Overweight | | | | Obesity | | | |
|---|---|---|---|---|---|---|---|---|---|---|---|---|---|
| Sex | Period | N per year[1] | Age-stand. rate[2] | HR[3] | 95% CI | N per year[1] | Age-stand. rate[2] | HR[3] | 95% CI | N per year[1] | Age-stand. rate[2] | HR[3] | 95% CI |
| males | 1972–1981 | 1.4 | 20.7 | 0.51 | (0.18–1.42) | 1.5 | 21.9 | 0.34 | (0.15–0.75) | 0.4 | 25.6 | 0.71 | (0.19–2.7) |
| | 1982–1991 | 2.8 | 26.8 | 0.53 | (0.21–1.32) | 4.7 | 36.5 | 0.5 | (0.26–0.94) | 1.9 | 54.5 | 1.41 | (0.56–3.55) |
| | 1992–2001 | 1 | 18.9 | 0.67 | (0.3–1.5) | 3.2 | 60.5 | 0.88 | (0.54–1.41) | 1.6 | 56.1 | 1.58 | (0.8–3.11) |
| | 2002–2011 | 4 | 56 | 1.48 | (0.93–2.35) | 5.8 | 40.7 | 0.82 | (0.57–1.17) | 4.1 | 80.3 | 1.56 | (0.95–2.57) |
| | 2012–2015 | 10.2 | 33.5 | reference, 1 | | 19.9 | 51.8 | reference, 1 | | 7.7 | 46.9 | reference, 1 | |
| | AAPC[4] (unadjusted) | - | - | 1.62 | (0.24–3.01) | - | - | 1.03 | (-0.07–2.14) | - | - | 1.07 | (-0.61–2.79) |
| | AAPC[4] (adjusted[3]) | - | - | 2.35 | (-0.53–5.31) | - | - | 1.8 | (-0.28–3.93) | - | - | 0.11 | (-2.78–3.1) |
| females | 1972–1981 | 0.5 | 10.1 | 0.2 | (0.06–0.62) | 1.6 | 31.5 | 0.46 | (0.2–1.09) | 1.1 | 23.6 | 0.46 | (0.15–1.39) |
| | 1982–1991 | 3.4 | 28.8 | 0.6 | (0.28–1.28) | 3.6 | 25 | 0.45 | (0.22–0.93) | 2.6 | 24.9 | 0.56 | (0.22–1.46) |
| | 1992–2001 | 2.3 | 38.1 | 0.94 | (0.54–1.66) | 2.6 | 39.7 | 1.03 | (0.59–1.81) | 1.4 | 37.1 | 0.8 | (0.4–1.61) |
| | 2002–2011 | 3.9 | 29.8 | 0.7 | (0.46–1.06) | 4 | 27.8 | 0.81 | (0.53–1.24) | 3 | 42.7 | 0.81 | (0.5–1.32) |
| | 2012–2015 | 17 | 41.8 | reference, 1 | | 15.4 | 36.1 | reference, 1 | | 10.8 | 41.3 | reference, 1 | |
| | AAPC[4] (unadjusted) | - | - | 1.97 | (0.66–3.3) | - | - | 0.96 | (-0.23–2.16) | - | - | 1.44 | (-0.01–2.91) |
| | AAPC[4] (adjusted[3]) | - | - | 2.35 | (-0.23–4.99) | - | - | 2.32 | (-0.14–4.84) | - | - | 2.03 | (-0.99–5.15) |

[1] Average annual number of colorectal cancers, [2]Adjusted for age, rates are comparable between calendar periods and sex, [3] Adjusted for age and time since study adjusted by age, education level, cigarette smoking, alcohol consumption, physical activity, height, and DM. [4] Average annual percentage change during 1972–2015.
